# The iBeetle large-scale RNAi screen reveals gene functions for insect development and physiology

**DOI:** 10.1038/ncomms8822

**Published:** 2015-07-28

**Authors:** Christian Schmitt-Engel, Dorothea Schultheis, Jonas Schwirz, Nadi Ströhlein, Nicole Troelenberg, Upalparna Majumdar, Van Anh Dao, Daniela Grossmann, Tobias Richter, Maike Tech, Jürgen Dönitz, Lizzy Gerischer, Mirko Theis, Inga Schild, Jochen Trauner, Nikolaus D. B. Koniszewski, Elke Küster, Sebastian Kittelmann, Yonggang Hu, Sabrina Lehmann, Janna Siemanowski, Julia Ulrich, Kristen A. Panfilio, Reinhard Schröder, Burkhard Morgenstern, Mario Stanke, Frank Buchhholz, Manfred Frasch, Siegfried Roth, Ernst A. Wimmer, Michael Schoppmeier, Martin Klingler, Gregor Bucher

**Affiliations:** 1 Johann-Friedrich-Blumenbach-Institut, GZMB, Georg-August-Universität Göttingen, Justus-von-Liebig-Weg 11, 37077 Göttingen, Germany; 2 Department Biologie, Entwicklungsbiologie, Friedrich-Alexander-Universität Erlangen-Nürnberg, Staudtstraße 5, 91058 Erlangen, Germany; 3 Institut für Entwicklungsbiologie, Universität zu Köln, Zülpicher Straße 47b, 50674 Cologne, Germany; 4 Abteilung Bioinformatik, Institut für Mikrobiologie und Genetik, Georg-August-Universität Göttingen, Goldschmidtstr. 1, 37077 Göttingen, Germany; 5 Abteilung für Bioinformatik, Universitätsmedizin Göttingen, Goldschmidtstr. 1, 37077 Göttingen, Germany; 6 Institut für Mathematik und Informatik, Ernst Moritz Arndt Universität Greifswald, Walther-Rathenau-Straße 47, 17487 Greifswald, Germany; 7 TU Dresden, Medical Faculty Carl Gustav Carus, Medical Systems Biology, Fetscherstr. 74, 01307 Dresden, Germany; 8 Eupheria Biotech GmbH, Dresden, Germany; 9 Abteilung für Genetik, Universität Rostock, Albert-Einsteinstr. 3, 18059 Rostock, Germany

## Abstract

Genetic screens are powerful tools to identify the genes required for a given biological process. However, for technical reasons, comprehensive screens have been restricted to very few model organisms. Therefore, although deep sequencing is revealing the genes of ever more insect species, the functional studies predominantly focus on candidate genes previously identified in *Drosophila*, which is biasing research towards conserved gene functions. RNAi screens in other organisms promise to reduce this bias. Here we present the results of the iBeetle screen, a large-scale, unbiased RNAi screen in the red flour beetle, *Tribolium castaneum*, which identifies gene functions in embryonic and postembryonic development, physiology and cell biology. The utility of *Tribolium* as a screening platform is demonstrated by the identification of genes involved in insect epithelial adhesion. This work transcends the restrictions of the candidate gene approach and opens fields of research not accessible in *Drosophila*.

## Abstract (web summary)

Unbiased screening for insect gene function has been largely restricted to *Drosophila*. Here, Schmitt-Engel *et al.* perform an unbiased large-scale RNAi screen in the red flour beetle *Tribolium castaneum* to identify putative gene functions.

## Graphical abstract

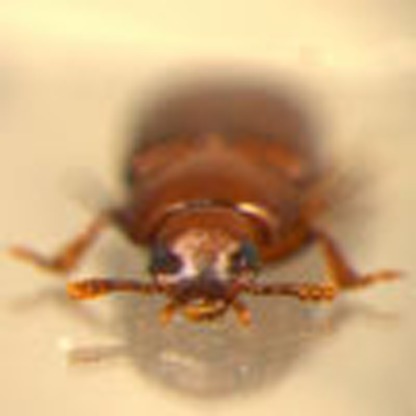

The importance of *Drosophila melanogaster* as a model system is in large part due to its amenability to elegant genetic screens, which allow the comprehensive identification of genes required for a given biological process^1^,^2^. Recently, unbiased genetic screens have also been performed in a few other insects, for example, the hymenopteran *Nasonia vitripennis* and the beetle *Tribolium castaneum*^3^,^4^,^5^,^6^. However, technical constraints prohibit saturation screens in these species. Hence, most of what we know about insect gene function remains based on *Drosophila* work. RNA interference (RNAi) has emerged as an alternative tool to knockdown gene function and has thus far been used for genome-wide screens in the nematode *Caenorhabditis elegans*, in *Drosophila* and in cell culture^7^,^8^,^9^,^10^,^11^.

In recent years, reverse genetics based on deep sequencing and RNAi has enabled functional investigations in ever more insect species, broadening the range of biological phenomena that can be analysed. However, the candidate gene approach still prevails: genes are selected based on previous findings in *Drosophila* or other model systems^12^,^13^,^14^,^15^,^16^,^17^,^18^,^19^,^20^,^21^. Consequently, the field of insect functional genetics suffers from several limitations. First, the candidate gene approach leads to a bias towards the study of conserved gene functions. Second, it has remained difficult to identify genes required for processes that are not represented in *Drosophila*. Finally, technical limitations and lineage specific gene losses or duplications prohibit the identification of comprehensive gene sets for a particular process in any single species. An unbiased, large-scale RNAi screen in a non-dipteran insect species should overcome many of these limitations.

The red flour beetle, *T. castaneum*, is well suited for this aim. In many respects its biology is more representative for insects than *Drosophila,* as its segmentation proceeds from a posterior growth zone, the larval head is not involuted and its extraembryonic tissues are well developed. Further, its modes of oogenesis and metamorphosis resemble those of other non-dipteran insects^22^,^23^,^24^,^25^,^26^,^27^,^28^. Further, *Tribolium* is a representative of the most species-rich animal taxon on earth, the coleopterans (beetles), including many devastating pests^29^. Finally, *Tribolium* research builds on an expanding transgenic toolkit^6^,^30^,^31^,^32^ and a particularly strong systemic RNAi response. Knockdown phenotypes can be induced at all life stages through dsRNA injection into the body cavity, and the RNAi effect spreads throughout the animal and is transferred to the offspring, often phenocopying null mutants^33^,^34^,^35^,^36^.

Here we present the results of the *iBeetle* screen, where we used RNAi targeting about 5,000 genes to identify novel gene functions during oogenesis, embryogenesis and metamorphosis in an unbiased way. We show that this screen has the power of overcoming the current limitations imposed by the candidate gene approach by the identification of unexpected novel players required for long studied processes. For instance, we describe the first bicaudal phenotype in *Tribolium* elicited by knockdown of the homeobox gene *Tc-homeobrain*; a gene so far not related to anterior–posterior axis formation. Further, we show that the unbiased detection of gene function in *Tribolium* allows opening new fields of research. For instance, many insects have odoriferous glands used for communication and defence but such glands are missing in *Drosophila*. In the screen, a set of genes were identified, which are involved in producing the defensive chemicals of the *Tribolium* odoriferous glands. Importantly, many of these genes were not identified in a recent RNA-seq approach confirming the power of a phenotypic screen. Finally, we show that *Tribolium* is an excellent alternative screening platform, where insect gene functions are efficiently identified. One example is the gene *Tc-Rbm24*, which we found to be required for muscle development in *Tribolium*. *Drosophila* does not have a respective ortholog while the vertebrate ortholog has recently been shown to be involved in muscle formation. As second example, we identified novel genes required for epithelial adhesion in *Tribolium*, the orthologs of which are required in *Drosophila* as well but had not been discovered in *Drosophila* screens.

## Results

### Design of the iBeetle screen

We developed a procedure that allowed efficient screening of several biological processes. Two screens were performed in parallel by injection of different developmental stages. In the ‘pupal injection screen', injected *pig19* transgenic female pupae (somatic muscles marked with EGFP) were scored for late metamorphosis phenotypes and, upon maturation to adults, their offspring embryos were analysed for muscle and cuticle phenotypes as readouts for defects in embryogenesis. This treatment knocked down both maternal and zygotic transcripts in developing embryos. In case of reduced egg production, ovaries of the injected females were analysed for oogenesis defects.

In the ‘larval injection screen', penultimate instar larvae (L6) were injected. Female larvae were derived from a cross between *D17Xred* (adult flight muscles marked with EGFP; X-linked DsRed marker allowed sexing of larvae) and *pearl* (white eyed) strains. Muscle phenotypes were scored during the pupal stage, and general morphological defects both at pupal and at adult stages. Ovaries were dissected and analysed whenever egg production was found to be strongly reduced. Finally, adult odoriferous glands were scored for alterations in size or colouring and dissected for closer inspection. Importantly, the larval injection screen allowed the identification of gene functions during metamorphosis without affecting essential functions during embryogenesis that would prevent analysis of later stages. We screened 5,300 genes in the (ongoing) pupal and 4,480 genes in the larval injection screen and present analyses of 3,400 genes included in both screens.

The iBeetle screen was designed as a first pass screen wherein each experiment was performed once, and off-target controls were done only for selected genes. We aimed at minimizing false negative annotations with the trade-off of an increased false positive rate. The genes to be knocked down were selected randomly, except that their annotations were based on RNA sequence data, which may have led to some enrichment of highly expressed genes (see Supplementary Table 1). Using the DEQOR prediction algorithm^37^, templates were selected for high RNAi efficiency and a low number of possible off-target sites.

dsRNA fragments with an average length of 479 bp were injected at a concentration of 1 μg μl^−1^. Phenotypes were annotated in an online database according to the EQM system (entity, quality, modifier)^38^ and using a controlled vocabulary based on the *Tribolium morphological ontology*^39^. In addition, the penetrance of phenotypes was recorded, and pictures and free text fields were used for further documentation (see Methods section for details). All abovementioned data sets of the larval and pupal injection screen in addition to sequence and orthology information of the entire *Tribolium* gene set are available at http://ibeetle-base.uni-goettingen.de^40^

### Tests for Sensitivity and Reproducibility

**Figure 1:**
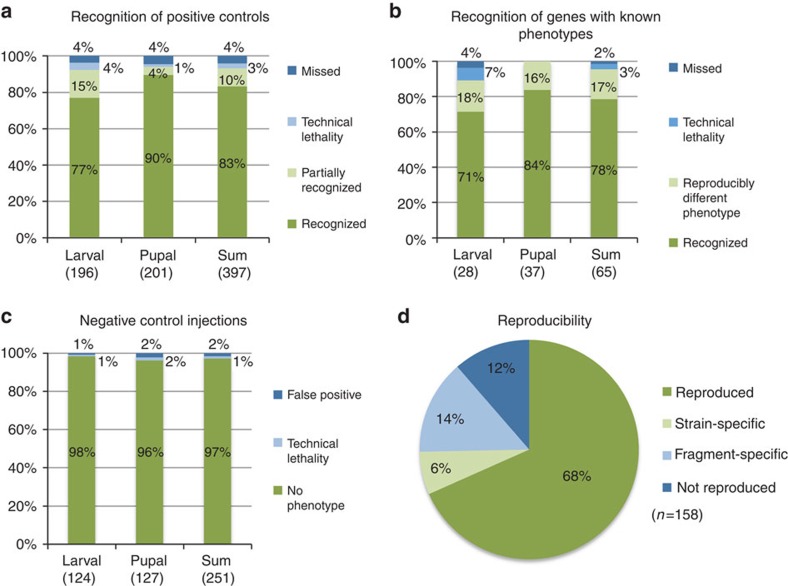
Sensitivity and reproducibility. (**a**) Recognition rates of 41 different positive controls shown separately for the larval and pupal injection screens (left and middle bars) and for both together (right bar). About 80% of the positive controls were fully recognized while another 10% were ‘partially recognized' (that is, not all phenotypic aspects were annotated). Only 4% of the positive controls were missed. ‘technical lethality': Expected phenotype not recognized owing to lethality of the animals for example, by injection. (**b**) Recognition rates for dsRNAs targeting 48 genes with published phenotypes, which had by chance been included in the screen. Of all, 78% were recognized with the published phenotype while 17% were annotated with a ‘reproducibly different phenotype'; that is, the differing phenotype was reproduced in independent experiments under iBeetle conditions. Hence, these different phenotypes are biologically meaningful and reflect that the timing and the degree of gene knockdown influences the phenotype. See Supplementary Note 1 for discussion of these cases. (**c**) Only 2% of all buffer injections led to false positive annotations. (**d**) A total of 158 dsRNAs were tested in independent injections with non-overlapping fragments. When the phenotype differed from the screening result, we analysed whether it was a false positive (‘not reproduced'), or whether the genetic background was the reason for the difference (strain specific). Finally, we tested whether the outcome depended on the dsRNA fragment used (fragment specific), which indicated off-target effects or splice variant specific knockdown.

To assess the sensitivity of our screen, roughly 5% of screened genes were positive controls from a set of 41 different genes (Supplementary Fig. 1). In addition, 48 previously published genes that happened to be within our gene set served as additional blind positive controls. Ninety-three per cent of the selected controls and 95% of the previously published genes were identified during the screen (Fig. 1a,b; Supplementary Table 2), which is similar to the findings in a genome-wide RNAi screen in *C. elegans*^11^. Interestingly, we found reproducibly different or additional phenotypes for 17% of the published genes, which likely reflects the dependence of the RNAi effect on injection time and dsRNA concentration (see Supplementary Note 1). As expected, negative control injections usually produced no phenotype (Fig. 1c; Supplementary Note 1; Supplementary Fig. 2).

To test for reproducibility, we repeated the screening procedure for 158 genes with high penetrance phenotypes (Fig. 1d; see Supplementary Data 1 for details). Seventy-four per cent of the phenotypes were reproduced (Fig. 1d). Notably, 6% of those phenotypes turned out to depend on the genetic background. This is in line with the emerging view that strain specificity of phenotypes may be more prevalent than appreciated previously^41^,^42^. Twelve per cent of the re-tested phenotypes turned out to be false positives, while 14% could be reproduced by the original dsRNA fragment but not with a non-overlapping fragment. The latter finding probably reflects off-target effects but in some cases may reflect biologically meaningful differences due to the isoform specific nature of RNAi knockdown in *Tribolium*^43^. Overall, the proportion of off-target effects using systemic RNAi with long dsRNA fragments in *Tribolium* was similar to that observed with short transgenic hairpin constructs in *Drosophila*, where 13% (*n*=9) of the constructs induced unexpected lethality^44^, but it was significantly lower than the 28% (*n*=18) or 24% (*n*=65) found with long hairpin constructs^8^,^45^. Considering 12% of ‘not reproduced' phenotypes and a maximum of 14% off-target effects, the maximum false positive rate in the iBeetle screen is 26%. Hence, the phenotypes detected in our first pass screen need to be confirmed by a second assay using non-overlapping fragments.

Reproducibility highly depended on the biological process scored. For lethality or wing blister phenotypes the reproducibility was >95%, while for L1 cuticle phenotypes it was about 60% (see Supplementary Fig. 3 for numbers for different phenotype classes).

To test in how far we missed the strong phenotypes due to incomplete knockdown, we injected dsRNAs targeting 98 genes at concentrations of 1 and 3 μg μl^−1^, and compared the phenotypic quality and strength. We found that phenotype strength was comparable for 86% of these genes.

### Essential genes identified in the larval and pupal screens

**Figure 2:**
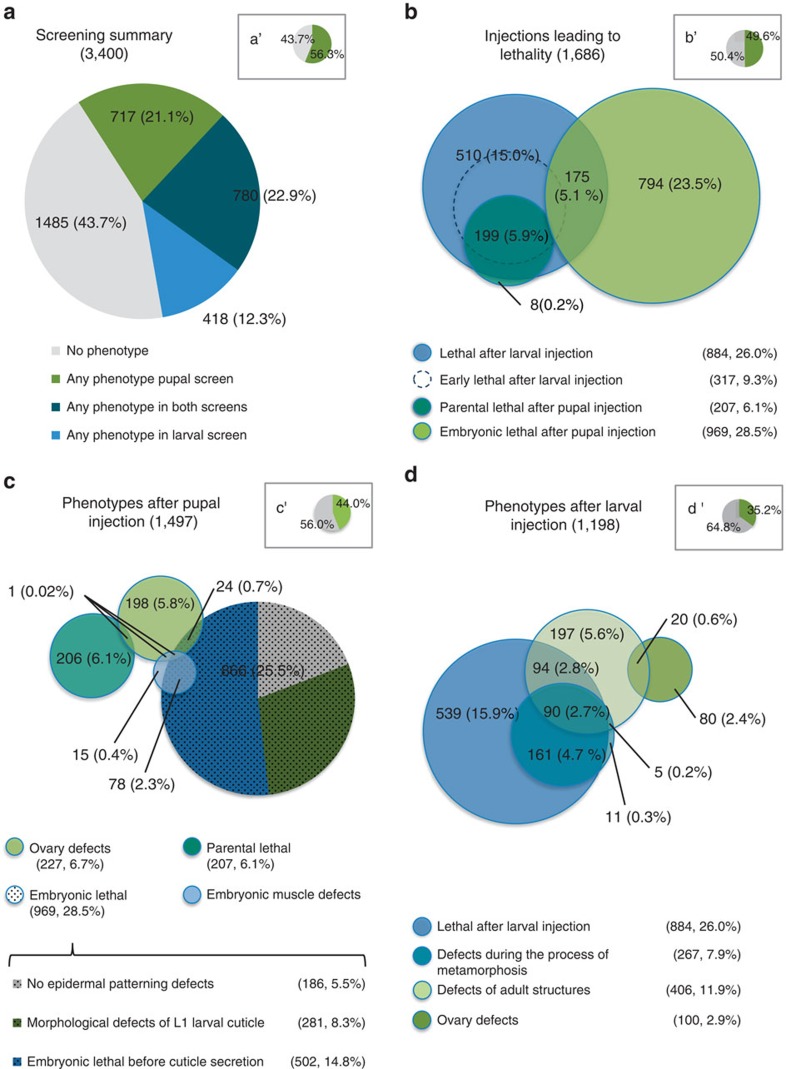
Essential and lethal genes. (**a**) For more than 56% of the injected genes, phenotypes were observed. The pupal injection screen revealed phenotypes for a larger portion of genes compared with the larval injection screen. (**b**) Death of the injected animals was scored 22 days post injection (larval injection; blue circle) and 11 days post injection (pupal and larval injection; dark green and hatched blue circles). Note that embryonic lethality is based on maternal and zygotic gene knockdown. ‘Parental lethal': death of the injected animal. (**c**) Selected phenotypic categories after pupal injection. Embryonic lethal injections are further categorized showing that more than half of the embryonic lethal genes lead to abortion of embryogenesis before cuticle secretion. (**d**) Phenotypic categories after larval injection. ‘Defects during the process of metamorphosis': metamorphosis not completed or entered precociously. Insets: relations to the entire data set.

Of the 3,400 genes tested in both the larval and pupal screens, 56.3% gave any phenotype (Fig. 2a), with 49.6% being lethal for at least one developmental stage (Fig. 2b). In all, 22.9% of the genes displayed a phenotype in both screens, while almost twice as many genes showed a phenotype exclusively in the pupal screen (21.1%) compared with phenotypes restricted to the larval screen (12.3%; Fig. 2a). Thirteen per cent of the genes showing any phenotype (including lethality) were beetle specific genes (Supplementary Table 1), illustrating the importance of screens in additional model organisms (see Supplementary Table 3 for definition of phenotype classes and Supplementary Data 2 for lists of treatments in the respective classes).

Basically, all genes required for survival to adulthood after pupal injection (5.9% of all genes; Fig. 2b) were also required after larval injection, likely due to housekeeping functions. The set of larval lethal genes appeared to be much larger (26% of all genes; Fig. 2b). However, this difference is in large parts due to low-penetrance lethal phenotypes, which were more likely to reach the threshold in the larval screen, where lethality was checked 22 days post injection instead of 11 days post injection in the pupal screen.

Embryonic lethality was found for 28.5% of all genes following knockdown of maternal and zygotic gene function (Fig. 2b,c). Altogether, 5.5% did not show obvious cuticle defects, while 8.3% displayed cuticle aberrations (Fig. 2c); 14.8% of all genes did not develop a cuticle, leading to a so called ‘empty egg phenotype'. This phenotypic class comprises genes with diverse essential functions, including housekeeping, cuticle formation itself or fertilization; however, some early patterning genes are also known to result in death before cuticle formation in *Tribolium*^46^,^47^,^48^. Further analyses using molecular markers are required to determine which process was affected for a given empty egg phenotype. Interestingly, 5.6% of all genes in the larval injection screen led to alterations of adult morphology without affecting larval survival or fertility, making them interesting candidates for understanding how adult morphologies develop and how they evolved (Fig. 2d).

### Comparison of embryonic and postembryonic patterning

**Figure 3:**
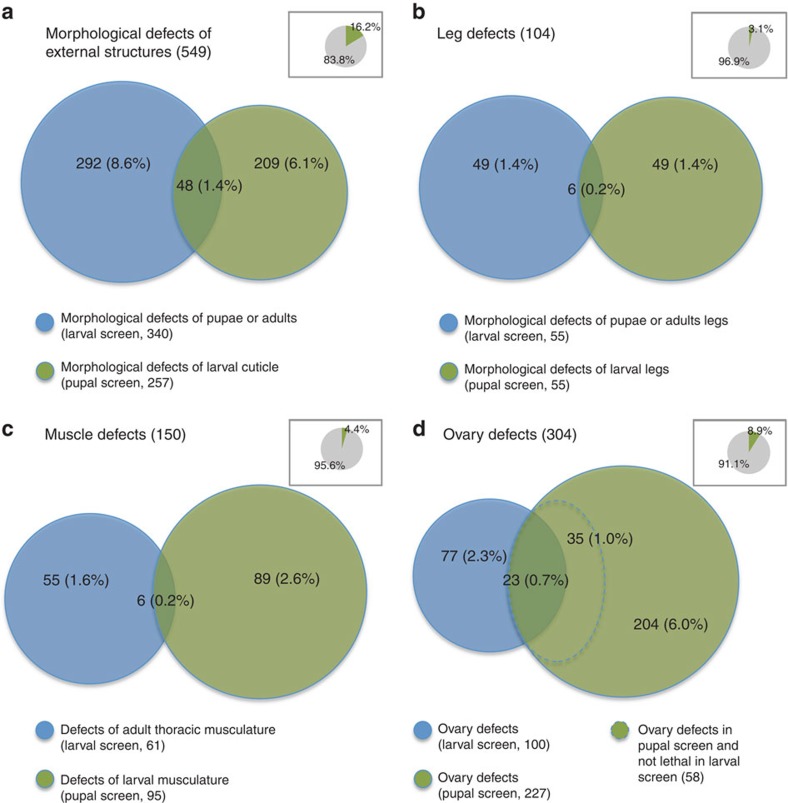
Comparison of gene sets involved in embryonic versus postembryonic development. (**a**) The gene sets required for cuticle morphology (that is, epidermal patterning) during embryogenesis and typical insect metamorphosis are largely non-overlapping. This indicates that patterning principles may differ to quite some extent between these two stages of major morphological change. (**b**,**c**) This observation also holds true for the subsets affecting leg morphology (**b**) and GFP marked somatic muscles (**c**) indicating that both ectodermal and other patterning processes differ. (**d**) Gene sets required for ovary function. Many genes required for egg production in the pupal injection screen (green circle) were lethal in the larval injection screen. Hence, reduced egg production for these genes was probably due to starvation (green area outside hatched line). When comparing the non-lethal treatments (blue circle and green circle with hatched blue outline) the number of genes with an ovary phenotype in the pupal and larval injection screen are more similar. Insets: relations to the entire data set.

For the first time our data allow the systematic comparison of the gene sets required for embryonic and postembryonic development in an insect with typical metamorphosis. Importantly, larval cells are largely re-used to form the adult epidermis in most insects, instead of being replaced by imaginal cells as is the case in *Drosophila*^24^. Nevertheless, the gene sets involved in embryonic and postembryonic patterning turned out to be largely non-overlapping (Fig. 3a). This is true for processes as different as leg or muscle development (Fig. 3b,c). In the case of oogenesis, the respective numbers are probably an overestimation because most genes leading to reduced egg production in the pupal screen were lethal in the larval screen (Fig. 3d). We assume that many of these apparent oogenesis phenotypes reflect incomplete knockdown of genes with basic physiological function because we found that many animals with reduced oogenesis showed a strongly reduced fat body. Subtracting these genes (those outside the dashed line in Fig. 3d), the overlap of genes with defects in both screening parts increases substantially. Together, these data reveal that development during typical insect metamorphosis partially relies on different mechanisms than during embryogenesis.

### Essential genes of *Tribolium* and *Drosophila*

The classical genetic screens for embryonic phenotypes in *Drosophila* revealed that about 5,000 genes were lethal when mutated (36% of the *Drosophila* protein coding genes), and for an additional 1,000 genes non-lethal phenotypes were identified. Hence, mutations in 43% of all loci revealed a phenotype of some kind^49^,^50^,^51^. The respective numbers for *Tribolium* are very similar (37 and 42%; all numbers in this paragraph are corrected assuming 26% false positives; see Methods section for calculations). However, in the iBeetle screen a much larger portion of the lethal genes is embryonic lethal compared with the *Drosophila* genetic screens (58% versus 20%). This is mainly due to a much higher number of embryonic lethal genes that show cuticle defects in *Tribolium* (81% versus 15% in *Drosophila*)^49^. One likely reason for this increased lethality is that in our screen we knocked down both maternal and zygotic gene functions, while in most *Drosophila* genetic screens only the maternal or zygotic contribution was affected^52^. As a consequence, in many cases zygotic functions were not detected because maternal contribution of gene products rescued zygotic mutations throughout *Drosophila* embryogenesis, leading to death after cuticle formation.

### Overcoming the candidate gene approach

**Figure 4:**
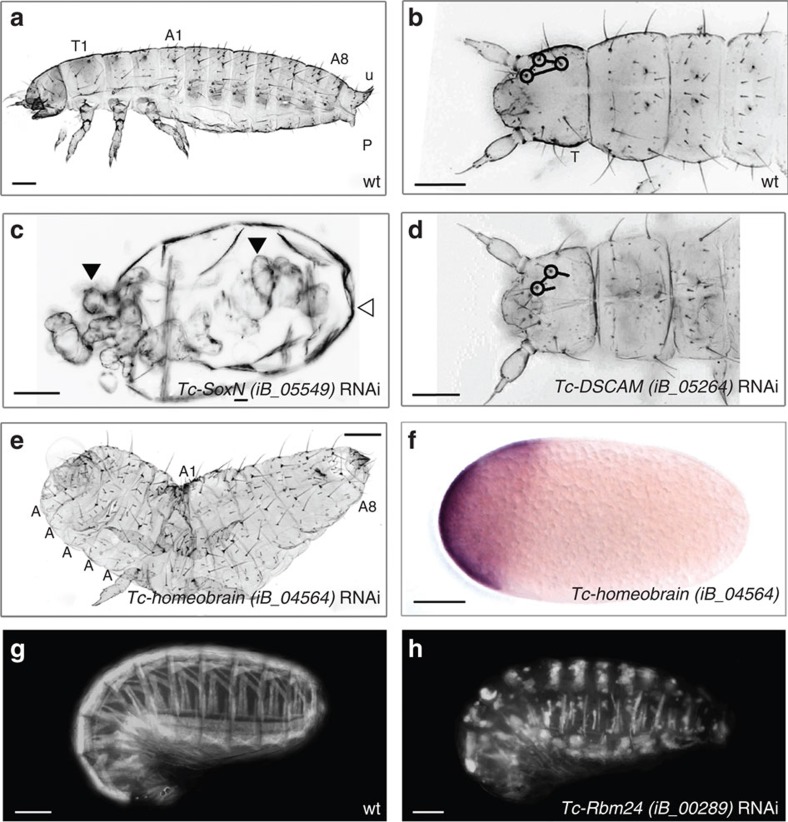
Embryonic phenotypes. (**a**,**b**) Wild-type L1 cuticles, with head setae marked by circles (**b**). T1: first thoracic segment; A1: first abdominal segment; A8: eighth abdominal segment; U: urogomphi; P: pygopods. (**c**) Unexpectedly, *Tc-SoxN* RNAi led to a strongly dorsalized cuticle phenotype without clear axes (embryo: filled arrowheads; vitelline membrane: open arrowhead). (**d**) *Tc-DSCAM* RNAi induced the deletion of head setae. (**e**) *Tc-homeobrain* RNAi caused a bicaudal phenotype (mirror image abdomina). This function is not known from *Drosophila homeobrain* and in *Tribolium* no bicaudal phenotype has been described before. (**f**) Early anterior zygotic expression of *Tc-homeobrain*. (**g**,**h**) *Tc-Rbm24* RNAi led to detached and shortened body wall muscles (wild type pattern in **g**). A muscle function is conserved in vertebrates while the ortholog was lost in *Drosophila*. Scale bars indicate 100 μm.

Most *Drosophila* segmentation genes have already been tested in *Tribolium*, revealing that a different gene set is involved in axis formation^46^,^53^,^54^. Overcoming this exhausted candidate gene approach, the iBeetle screen indeed identified novel players. For instance, knockdown of the homeobox gene *Tc-homeobrain* elicited a mirror image duplication of the abdomen similar to the *Drosophila* bicaudal phenotype and was found to be one of the earliest anteriorly expressed zygotic genes (Fig. 4a,e,f). No cuticle phenotype has been described for *Drosophila homeobrain,* and so far no *Tribolium* gene had been found to elicit a bicaudal phenotype. *SoxNeuro* is required for *Drosophila* central nervous system development^55^,^56^, but was not reported to be involved in cuticle patterning. In the iBeetle screen, *Tc-SoxNeuro* knockdown resulted in a dorsalized phenotype, suggesting that *Tc-SoxNeuro* influences early dorsoventral patterning (Fig. 4c). Another example is *Drosophila Dscam,* which is a cell adhesion gene with extensive alternative splicing and which is known to act in neurogenesis and immunity^57^,^58^. The iBeetle screen identified potential additional essential roles of *Tc-Dscam* in sensory organ formation (Fig. 4d).

### New fields of research

**Figure 5:**
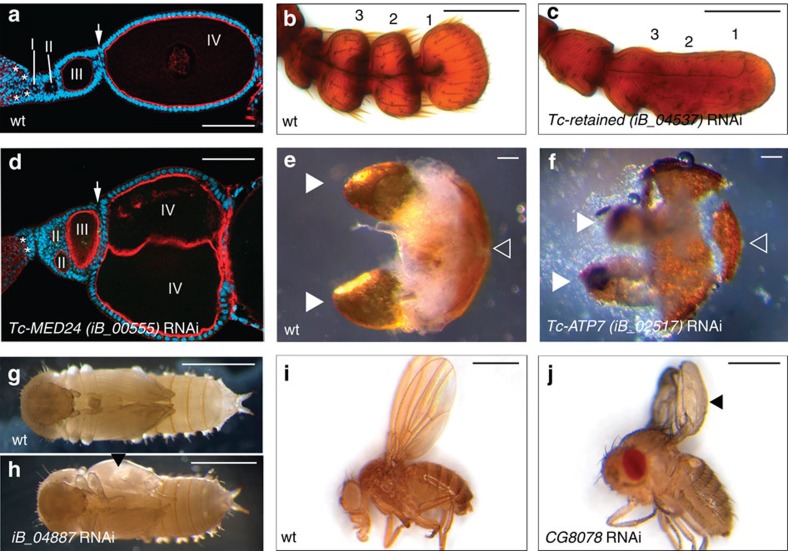
Postembryonic phenotypes. (**a**) Wild-type ovary stained for F-actin (red) and DNA (blue). Pro-oocytes (asterisks) become encapsulated by somatic follicle cells and separated by stalk cells (arrow). (**d**) Upon *Tc-MED24* RNAi, egg chambers are misarranged, not separated by stalk cells (arrow) and subsequently they fuse (IV). (**b**,**c**) After *Tc-retained-*RNAi the three most distal antennomeres (1–3) of the adult antenna are fused. (**e**,**f**) RNAi against *Tc-ATP7* led to strongly reduced odoriferous gland content and partially melanized secretions (white arrowheads; remnant of posterior abdominal cuticle marked by open arrowhead:). (**g**–**j**) The knockdown of *iB_04887* led to wing blisters in *Tribolium* pupae (arrowhead in **h**). Transgenic RNAi against the *Drosophila* ortholog showed the same phenotype, revealing a novel candidate for integrin mediated adhesion (arrowhead in **j**). Scale bars indicate 100 μm in (**a**–**f**) and 1 mm in (**g**–**j**).

Odoriferous stink glands play crucial roles in insect defence and communication but are not present in *Drosophila*^59^,^60^. In the iBeetle screen, we identified 32 genes with relevant phenotypes, including the absence of the gland contents, altered colour and composition of secretions, or melanosis. Interestingly, only 5 among these 32 genes showed an enrichment of greater than fourfold in odoriferous gland transcriptomes compared with mid-abdominal tissues^60^, illustrating that a phenotypic screen can only partially be replaced by transcriptomic approaches (see Supplementary Fig. 4). For instance, the *Tc-copper-transporting-ATPase-I* (*Tc-ATP7*) is neither upregulated nor downregulated (Supplementary Fig. 5) but RNAi mediated knockdown (iB_02517) caused a reduced gland content and melanosis phenotype (Fig. 5f) and a loss of benzoquinones (Supplementary Fig. 6). Another emerging field is the shaping of the adult body during typical holometabolous insect metamorphosis were larval epidermal cells are reused^24^. For instance, *Tc-retained* led to rounded female genitalia and the fusion of distal antennal segments (Fig. 5c). Interestingly, these anatomical features vary in Tenebrionids^61^, making this gene a good candidate for morphological evolutionary studies. Finally, one difference between *Tribolium* telotrophic oogenesis and *Drosophila* meroistic oogenesis is that in *Tribolium* germ line stem cells stop proliferating at larval stages while the somatic stem cell lineage remains active throughout life^23^. In *Drosophila*, both lineages remain active and dependent on each other, making it difficult to study the somatic lineage independently. In the iBeetle screen we identified several genes probably required for the somatic lineage, such as *Tc-MED24* whose knockdown led to incomplete separation of egg chambers and a reduced number of follicle cells (Fig. 5d).

### *Tribolium* as a screening platform

Wing blisters indicate the loss of adhesion between the two epithelial sheets of the wing blade. This phenotype has been used in *Drosophila* to identify components of integrin mediated adhesion^62^,^63^. We found 49 genes associated with wing blisters in our screen. Thirty-four were re-tested with non-overlapping fragments. All were confirmed in the screening strain but one led to lethality in another genetic background. Seventeen of these were previously annotated with GO terms connected to cytoskeletal function or adhesion, but 14 had unrelated functional annotations and 5 did not have any functional annotation at all^64^. We tested 19 genes in *Drosophila* by transgenic RNAi knockdown, using two different wing disc driver lines. Out of seven genes without previous annotations with respect to cell adhesion or cytoskeletal function in *Drosophila*, four uncovered a wing blister or ‘crumbled wing' phenotype, indicating an involvement in *Drosophila* epithelial adhesion (See Supplementary Table 4). One example is the *Tribolium* ortholog of CG8078, which is predicted to be involved in tRNA thio-modification and was not implicated in cell adhesion before (Fig. 5h,j). Notably, some wing blister genes known from *Drosophila* were not recovered in the iBeetle screen owing to larval lethality before wing development (see Supplementary Table 5). Therefore, we injected at a later stage (L7) and indeed, two additional genes showed the wing blister phenotype. Hence, a new screen focused on injections at later larval stages is likely to reveal additional wing blister genes. In summary, the *Drosophila* and *Tribolium* screening platforms appear to reveal different subsets of genes involved in a common cellular process, suggesting that the use of *Tribolium* as alternative screening platform may reveal novel players relevant for general insect biology.

We identified many known and novel genes required for muscle development. In *Tc-Rbm24* RNAi embryos, for instance, the muscles form small, round syncytia, which eventually seem to decay (Fig. 4h). The gene codes for an RNA binding protein of the RRM superfamily. Of note, it lacks an ortholog in *Drosophila* but vertebrate orthologs are active in myogenesis^65^,^66^. Hence, our data show the conservation of myogenic function of *Rbm24* in Bilateria.

## Discussion

Here we showed that the iBeetle RNAi screen achieved efficient and sensitive detection of novel gene functions at four developmental stages and in several processes in the red flour beetle *Tribolium*. The reproducibility varied depending on the phenotypic class. Highly penetrant phenotypes with simple readout (for example, wing blister, lethality) reproduced with more than 95% while the reproduction rate of embryonic developmental defects was significantly lower (about 60%). One reason is that iBeetle was designed as first pass screen without replicates while in cell based RNAi screens replicates and different reagents targeting the same gene function are usually used to minimize the portion of false positive data. Another important reason is that in *Tribolium* moderate knockdown of housekeeping genes during embryogenesis leads to abortion of embryogenesis at different stages leading to diverse cuticle defects. Hence, any off-target effect affecting a housekeeping gene is likely to result in diverse cuticle defects. Hence, it is advisable to focus on phenotypes annotated with high penetrance (>50%) and confirmation of phenotypes using non-overlapping dsRNA fragments is essential.

Our data show that a significant portion of insect gene functions becomes apparent when using additional model systems for several reasons. First, some aspects of insect biology are more representative in *Tribolium* compared with *Drosophila* like for instance metamorphosis based on the re-use of larval epidermal cells. Indeed, we identify novel genes associated with this process, the orthologs of which might have lost their function in *Drosophila* due to its derived mode of metamorphosis. Second, the different characteristics of the screening procedures may lead to detection of different subsets of the gene set involved in a conserved process. For instance, we identified novel genes leading to a wing blister phenotype but not all genes known from *Drosophila* were recovered in our screen. This suggests that neither system appears to have the power of detecting the comprehensive gene set of a given process. Finally, 13% of the genes associated with phenotypes in our screen do not have orthologs in *Drosophila* and therefore need to be studied in other model systems.

In summary, the iBeetle screen helps to overcome several current limitations in insect functional genetics. First, the candidate gene approach prevailing in emerging model organisms cannot reveal novel gene functions. This is superseded by the hypothesis independent identification of genes. Second, new biological processes can now be investigated, the genetic bases of which remained obscure because they are too derived or not present in the fly, or not amenable in the fly due to technical constraints. Third, we show that *Tribolium* is a powerful complementary screening platform for basic processes that are being studied in other organisms, such as epithelial adhesion. Importantly, the dsRNA template library generated in this project facilitates future screens focused on additional topics. Taken together, the iBeetle screen aids in bridging the gap between large-scale gene discovery by next generation sequencing and the small-scale approaches used for uncovering the function of novel genes.

## Methods

### Selection of genes and dsRNA production

The genes to be knocked down were selected at random. However, we required that the predictions were well supported by cDNA sequence data. Further, we excluded genes close to neighbouring same-strand gene predictions to avoid the double injection of genes, which were erroneously annotated as two genes. As the coverage by RNA-seq was low at project start, the gene set may have been enriched in genes expressed above average. This may explain the higher portion of conserved genes in the set of screened genes (82% in the iBeetle screen versus 59% in the official gene set; see Supplementary Table 1).

The target transcript sequences were analysed using the DEQOR algorithm to identify stretches with highest amount of sequences predicted to be efficiently recognized by the RNAi machinery and the lowest amount of potential off-target sites. Fragments within these stretches were amplified by PCR with gene-specific primers, which were tagged with parts of the T7 (3′ primer) and SP6 (5′ primer) promoter sequences (T7 tag 5′-CTCACTATAGGGAGA-3′; SP6 tag 5′-TGACACTATAGAAGTG-3′). The sequences are available at the iBeetle-Base ( http://ibeetle-base.uni-goettingen.de/) and in Supplementary Data 3. The products from these PCR reactions were used as templates in a second PCR using T7 and SP6-T7 primers to generate templates for bidirectional *in vitro* transcription using T7-RNA polymerase. (Note that due to the different tags on 5′ and 3′ end of the fragments, the iBeetle library can be used for the generation of *in situ* probes as well.) For quality control, all products of the second PCR were checked on a gel and sequenced. All dsRNAs were assessed for purity by gel electrophoresis, the concentration was measured by the Ribogreen Assay (Life Technologies), and adjusted to 1μg μl^−1^ using injection buffer. Template production was performed by Eupheria Biotech GmbH (Dresden). Overall, 7,200 *in silico* defined templates were channelled into the pipeline leading to the production of 6,147 templates of which 5,670 dsRNAs were produced and sent to the screening centres. The success rate was about 85% for template generation and 92% for dsRNA production, resulting in an overall success rate of about 79%. The average length of the templates was 479 bp.

### The screening procedure

Animals for 24 experiments were reared and processed in parallel using the equipment developed by Berghammer *et al.*^67^. For the injections two transgenic lines were used, where EGFP enhancer traps marked larval (*pig19*) or adult muscles (*D17*) in the pupal and larval injection screen, respectively (see Supplementary Fig. 7). An X-linked transgene insertion (*Xred*, expressing DsRed in larval eyes) was used for sexing larvae. dsRNA solution in the volume 1 μg μl^−1^ was injected into 10 animals per experiment using a FemtoJet express device (Eppendorf). It was injected as much as possible without interfering with survival. For injection and morphological inspection, larvae and adult beetles were anaesthetized with ice or carbon dioxide. Pupae were affixed to microscope slides for injection using a double sided sticky tape or rubber based cement (Fixogum). Inspection of pupae and adult morphology and phenotype documentation was performed with epifluorescence stereomicroscopes (Leica M205 FA). The data were documented electronically during analysis using an online interface. The interface allowed the documentation of technical remarks and offered dropdown lists with controlled vocabularies for documentation. In addition, pictures were uploaded for the documentation of the annotations and remained linked to the respective annotation. This allowed displaying the relevant pictures in the search results. For analysis of embryonic muscles, living embryos were dechorionated, embedded in Halocarbon oil (Voltalef 10S) and analysed using upright fluorescence microscopes (Zeiss, Jena). Fourty-one dsRNAs targeting genes with known phenotypes were randomly introduced as positive controls with a frequency of 1 or 2 per 24 injections. The first and last of the 24 injections performed on 1 day were negative control injections (buffer). The throughput of the screen was 21 and 28 genes per week in the pupal and larval injection screen, respectively. The workflow is shown in Supplementary Table 6; the schedules are shown in Supplementary Figs 8 and 9. All phenotypes shown in this paper were reproduced with non-overlapping fragments using the SB strain as a different genetic background (see Supplementary Table 7 for sequences). The data are available at http://ibeetle-base.uni-goettingen.de/40.

### GC–MS of odoriferous stink gland contents

dsRNA was injected in animals at mid-pupa stage (SB strain). Injected pupae as well as uninjected control pupae were kept on whole-grain flour at 32 °C. Ten days after hatching both the prothoracic and the abdominal glands of one beetle were dissected and crushed pairwise in 50 μl methanol (Merck Millipore: SupraSolv). The samples were stored at −20 °C and subjected to gas chromatography mass spectrometry (GC–MS) measurements within 48 h. A volume of 1 μl was loaded per sample by a split injector into a gas chromatograph (Agilent Technologies 6890N Network GC System) connected to a mass spectrometer (Agilent Technologies 5973 Network Mass Selective Detector). For chromatogram analysis the software MSD ChemStation D.02.00.275 (Agilent Technologies) was used.

### Comparison of essential genes

The portions of essential (that is, lethal) genes in *Drosophila* that had been published previously vary with the assumed total number of genes at the time. For our comparison, we related the published absolute numbers of essential genes to the number of protein coding genes of the current *Drosophila* genome release (6.02). For *Tribolium* we reduced the numbers observed in the iBeetle screen by the estimated portion of false positives in our data set (26%; see above). See Supplementary Table 8 for numbers and calculations.

## Additional information

**How to cite this article:** Schmitt-Engel, C. *et al.* The iBeetle large-scale RNAi screen reveals gene functions for insect development and physiology. *Nat. Commun.* 6:7822 doi: 10.1038/ncomms8822 (2015).

## Supplementary Material

Supplementary Information Supplementary Figures 1-9, Supplementary Tables 1-8, Supplementary Note 1 and Supplementary References

Supplementary Data 1 Details on tests for reproducibility, strain specificity, off target effects and effects of dsRNA concentration on phenotypic strength

Supplementary Data 2 Lists of treatments leading to certain phenotypic classes

Supplementary Data 3 dsRNA sequences used in the screen
