# Supplementary material for: The iBeetle large-scale RNAi screen reveals gene functions for insect development and physiology
Source: Nat Commun. 2015 Jul 28;6:7822. doi: 10.1038/ncomms8822 (PMC4525174; doi:10.1038/ncomms8822)
Supplement: Supplementary Information — Supplementary Figures 1-9, Supplementary Tables 1-8, Supplementary Note 1 and Supplementary References [file ncomms8822-s1.pdf]

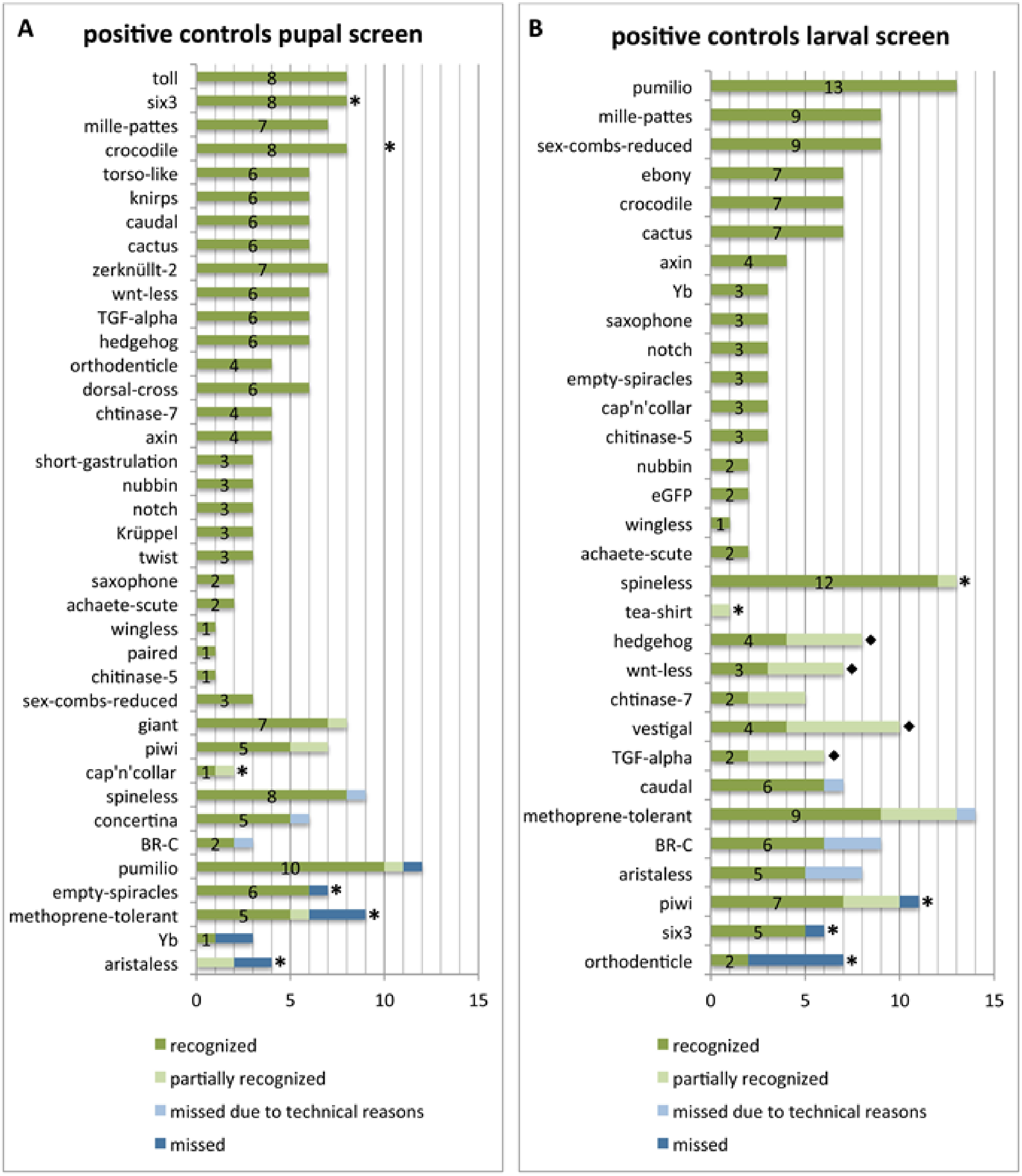

Supplementary Figure 1 | Recognition of positive controls

The length of each bar indicates how often the respective control was used in the screen, and the color code indicates how often it was fully (dark green) or partially identified (light green), and how often it was missed (blue colors).

Asterisks indicate controls with a very subtle phenotype. Black diamonds indicate positive controls with complex phenotypes, which were rated as “partially recognized” when three quarter or more of phenotypic aspects were correctly identified.

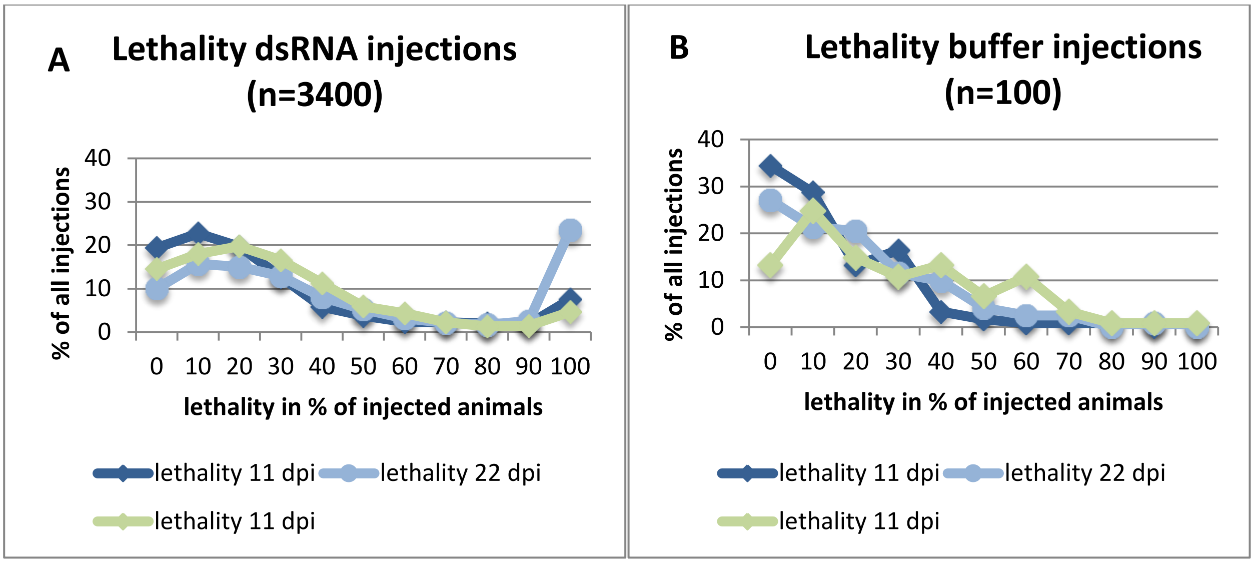

Supplementary Figure 2 | Lethality of injected animals after dsRNA and buffer injections in the pupal and the larval injection screens

A) Shown is the distribution of lethality rates of the injected animals in RNAi experiments. Lethality was documented at 11 and 22 days post injection (dpi) in the larval injection screen (dark and light blue) and at 11 dpi in the pupal injection screen (green). Most experiments showed a lethality of up to 30%. The distribution dropped up to 80% but increased again from 90% onwards. B) The same distributions shown for buffer injections. Here, the 90 and 100% values were not increased. Taken together, lethality rates below 80% were most likely “technical lethality” while higher lethality rates were probably the consequence of RNAi targeting an essential gene. Hence, we considered “lethality” as a phenotype only when at least 90% of the injected animals had died.

a

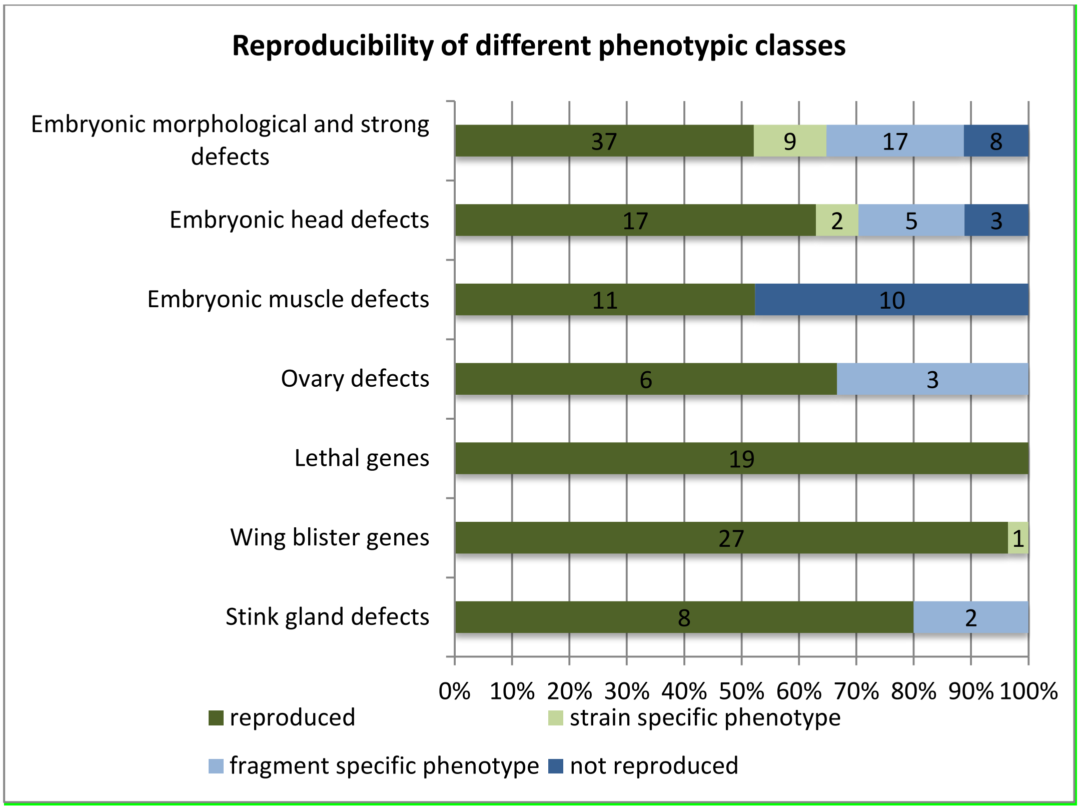

b

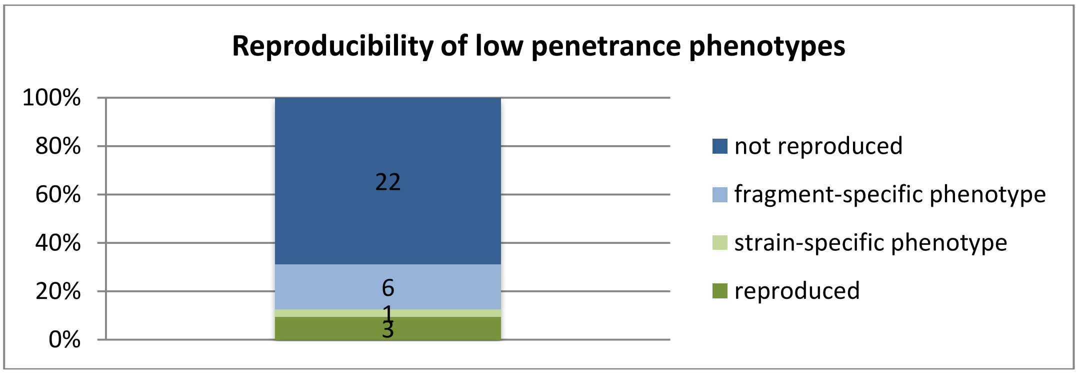

Supplementary Figure 3 | Reproducibility depends on the phenotype class
158 genes matching our criteria for significant phenotypes (see Supplementary Table 3) were tested for reproducibility. Where possible, a non-overlapping fragment was injected into a strain with different genetic background (SB in most cases). If the phenotype was not reproduced, the non-overlapping fragment was injected into the strain used in the screen. This allowed distinguishing whether the non-reproduced phenotypes were due to fragment- or strain-specific differences (i.e. putative off target effects or genetic background effects). a) Phenotypes of different processes with a penetrance > 50% were tested. Some phenotype classes like lethality and wing blistering were reproduced with very high frequency while embryonic phenotypes were more frequently not reproduced (see main text for discussion on likely reason). b) Phenotypes that were annotated with a penetrance <50% were frequently not reproducible.

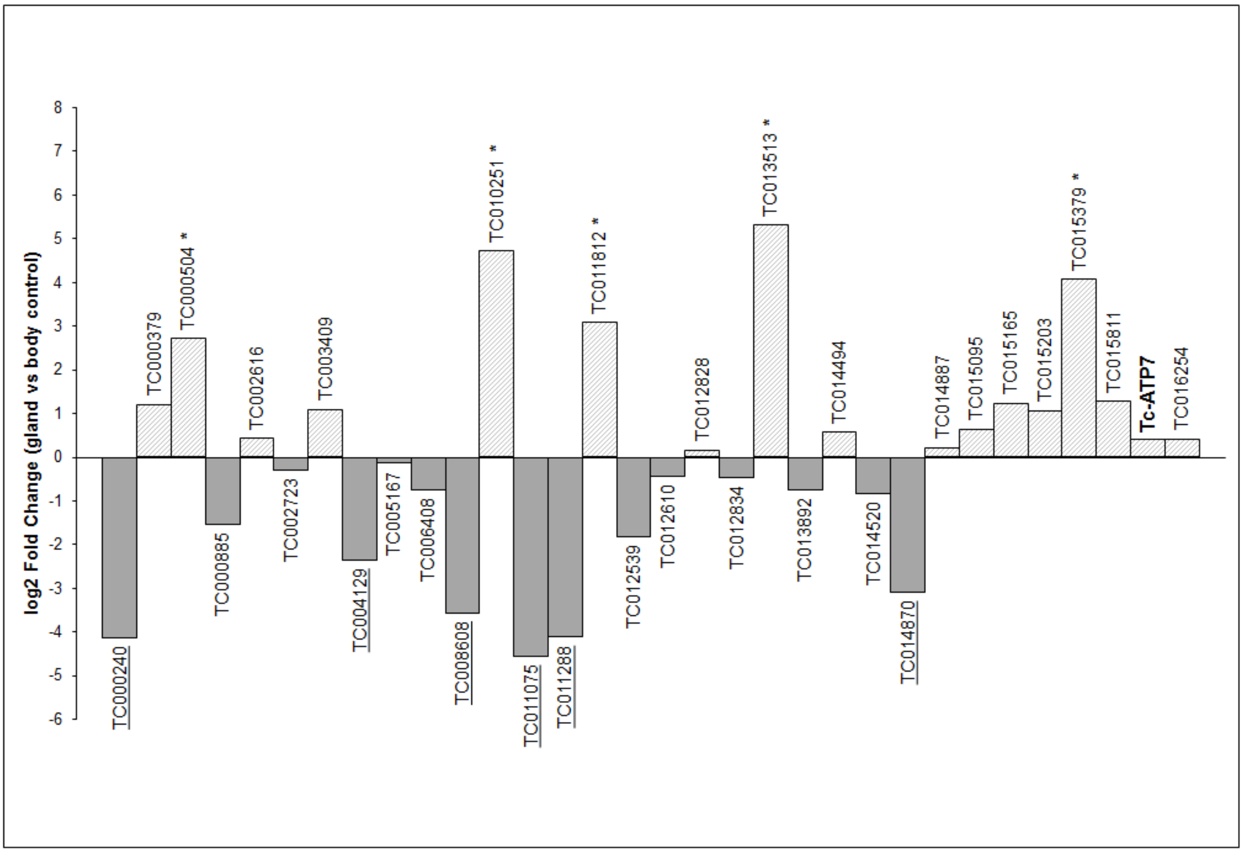

Supplementary Figure 4 | Differential expression of 32 iBeetle-identified gland phenotype-causing genes

We determined for the 32 genes with a confirmed odoriferous gland phenotype, whether the transcripts were enriched in the glands (data taken from Li et al. 2013)[1]. Each bar represents the level of expression of a gene in male abdominal glands relative to its expression in the mid-abdomen body control. Only five genes (marked with an asterisk) showed >4-fold enrichment in the odoriferous glands and would have been chosen as candidates based on a transcriptomics approach, thereby missing genes such as *Tc-ATP7* (bold) that is neither up nor down regulated but revealed a reduced gland content and melanosis phenotype upon knockdown. The iBeetle screen even identified genes causing a gland phenotype upon knock-down whose expression is strongly reduced in the odoriferous glands (underlined TC numbers). Thus, most genes detected in the iBeetle screen would not have been selected in an approach based on differential gene expression. Differences in the expression intensities are given as logarithm 2 fold change, calculated as log2 of the quotient [depth (gland) / depth (control)], where depth is calculated as number of reads multiplied with length of reads (38bp) divided by specific length of gene transcript.

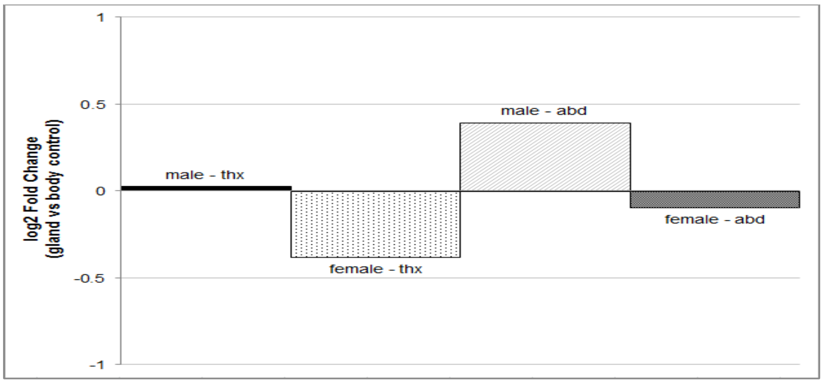

Supplementary Figure 5 | Differential expression of *Tc-ATP7* in different gland tissues

Expression of the copper-transporting ATPase *Tc-ATP7* in male and female thoracic (thx) and abdominal (abd) odoriferous glands is not significantly de- or increased relative to the expression in the mid-abdomen body control.

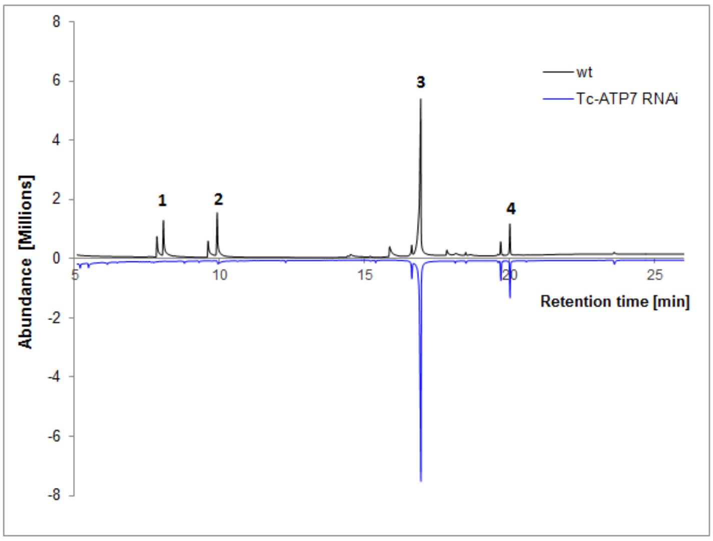

Supplementary Figure 6 | Gas chromatograms of stink gland contents.

The four main volatile substances detected via GC-MS in abdominal glands of wild type beetles (black line) are 1: 2-Methyl-1,4-benzoquinone; 2: 2-Ethyl-1,4-benzoquinone; 3: 1-Pentadecene; 4: 1-Heptadecene. After knockdown of Tc-ATP7 (blue line, for better comparison plotted as negative values) gland secretions lack the benzoquinones (peaks 1 and 2).

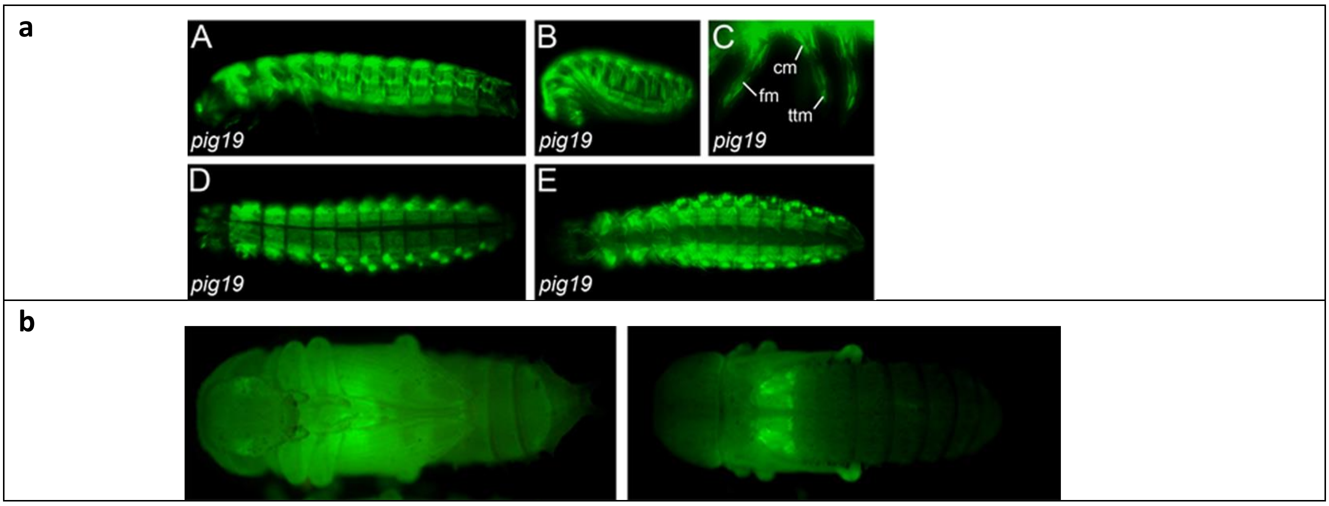

Supplementary Figure 7 | Enhancer trap lines used in the screen

a) Females carrying the *pig19* enhancer trap were used for pupal injections. In this enhancer trap line EGFP is expressed in somatic muscles including leg muscles. Upon maturation, injected female pupae were mated with male beetles of the *black* strain (dark cuticle)[2] in order to allow quick assessment of adult survival of injected females without the need for repeated adult sexing.

b) For larval injections we used daughters of a cross between pearl females [3] and males of the D17Xred strain. This line is carrying a Minos transposon [4] insertion (D17) containing the 3xP3-EGFP marker which has captured an adult flight muscle enhancer. In addition, a piggyBac transposon carrying DsRed coding sequence driven by a 6xP3-promoter was inserted on the X-chromosome. Hence, female larvae were identified as larvae expressing the DsRed fluorescent protein in the eyes and the brain. Injected females were crossed to black males.

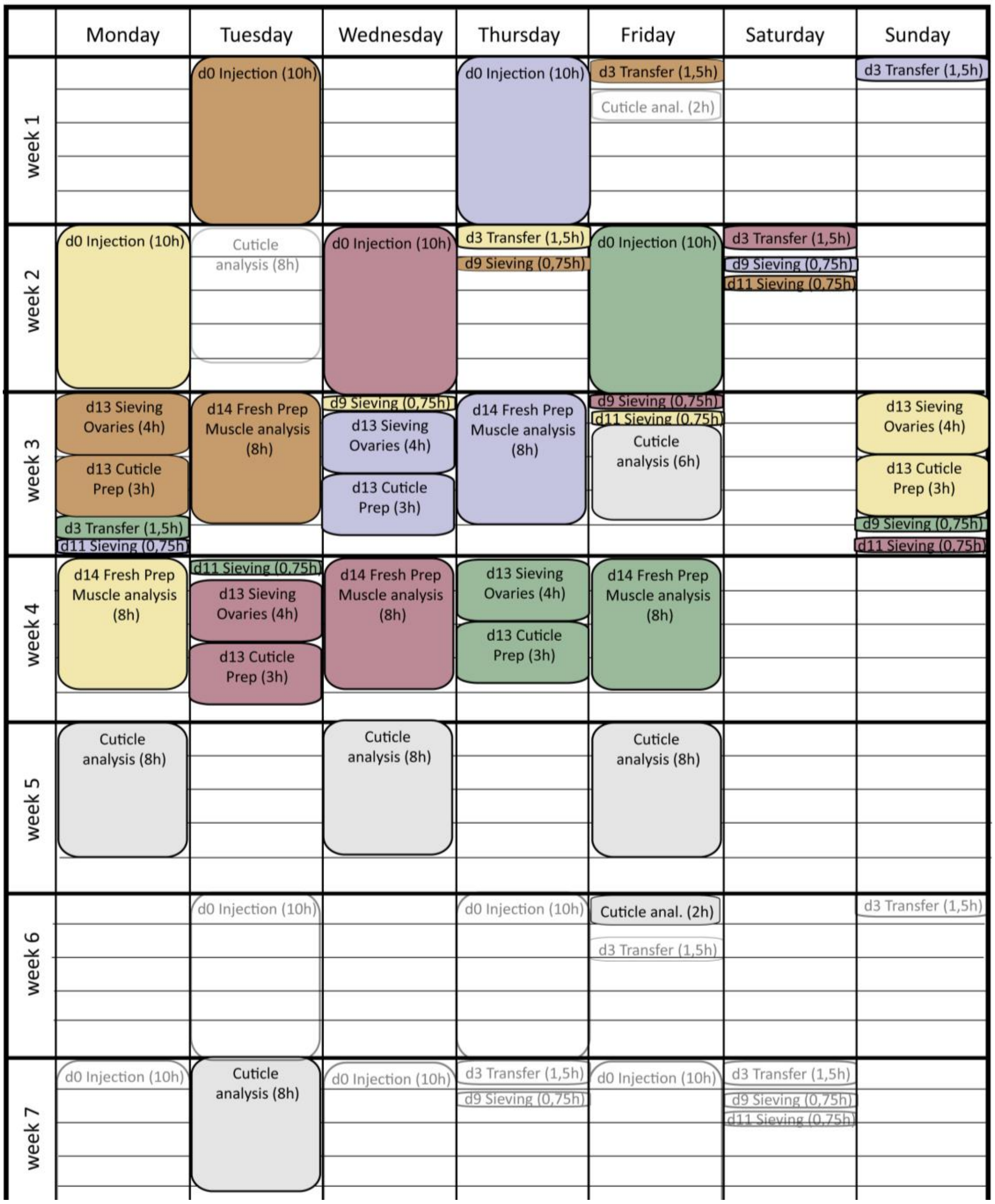

Supplementary Figure 8 | Schedule of the pupal injection screen

The five repetitions performed in parallel are shown in different colours. Each step of each repetition is shown at the day of processing and the approximate time required for this step is given. After five weeks, the same schedule was repeated (open boxes in week 6 and 7). The cuticle analysis can be performed at any time, because the cuticle preparations are stable over time.

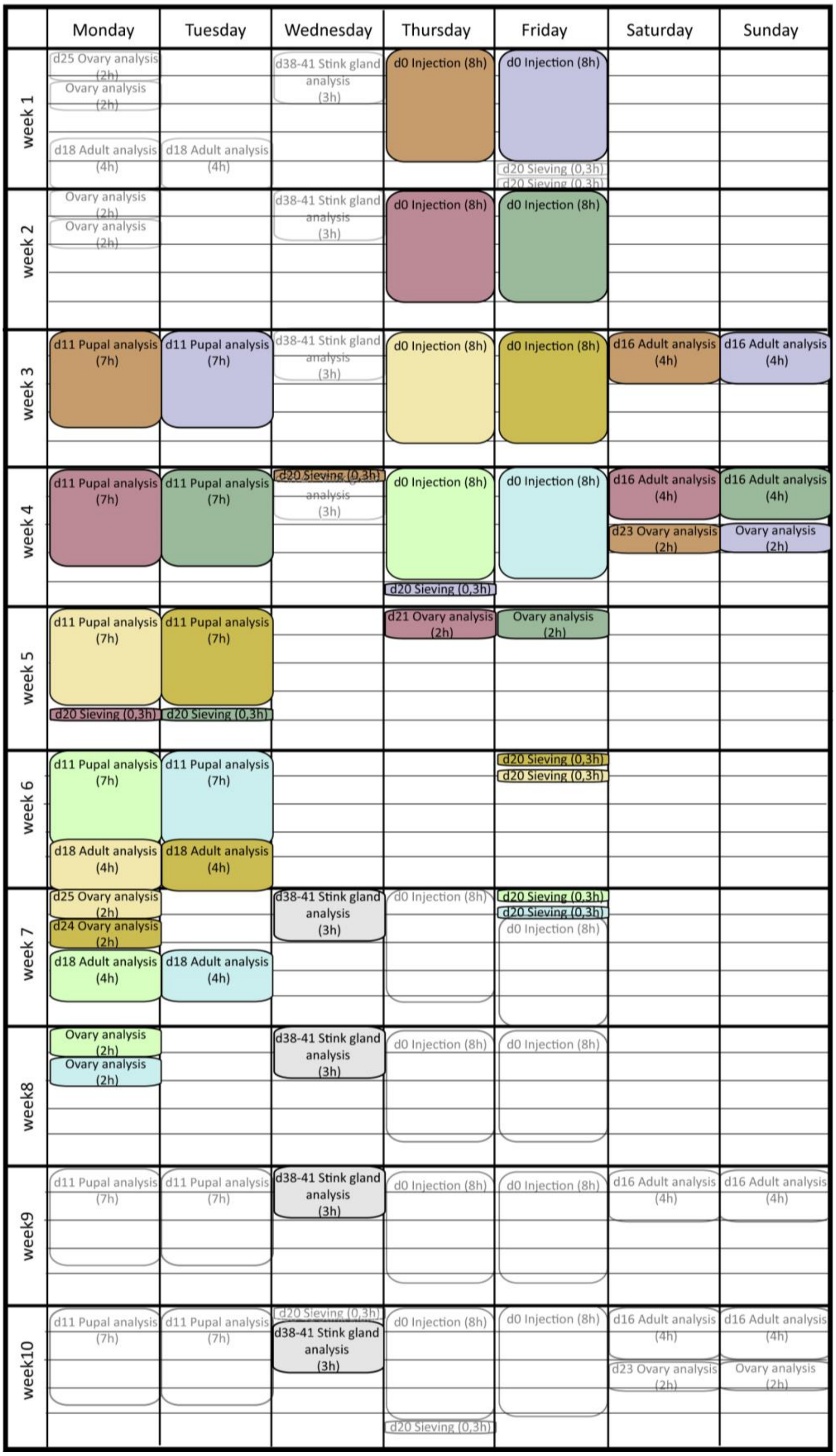

Supplementary Figure 9| Schedule of the larval injection screen

In the larval injection screen, eight repetitions were performed in parallel and the schedule repeated after 6 weeks.

Supplementary Table 1 | Comparison of iBeetle analysis set with official gene set

	Official gene set		iBeetle gene set		Genes with Phenotype in iBeetle		
	**N**	**%**	**N'**	**%**	**N''**	**%**	**% of iBeetle genes with phenotype (N''/N')**
**Genes**	16561	100	3400	100	1915	100	56
**Conserved**	9838	59	2784	82	1659	87	60
**Beetle specific**	6723	41	616	18	256	13	42
**Conserved in *Drosphila***	8334	50	2496	73	1546	81	62
**Lost in *Drosophila***	1505	9	288	8	113	6	39

"beetle specific": NCBI blast did reveal orthologs only in beetles;

"lost in *Drosophila*": NCBI blast revealed orthologs in other insects but not *Drosophila*

Note: the percentage in the rightmost column relates the values in column N'' to respective values in column N', e.g. 39%=113/288.

Supplementary Table 2| iBeetle results for genes with published or known phenotypes

Pupal injection screen

gene	iB-#	TC-#	screening result	reference
achaete-scute	iB_04489	TC008433	loss of larval bristles and setae	Wheeler et al. 2003, this study
axin	iB_04108	TC006314	as expected	Fu et al. 2012
broad-complex	iB_03960	TC005474	no phenotype, potentially due to alternative splicing	this study
cactus	iB_00322	TC002003	as expected	Fonseca et al. 2008; Roth unpublished
CG16778	iB_04153	TC006481	muscle defects, differs from earlier experiments where defects in embryonic head and abdominal development were found	Schoppmeier, unpublished
Delta	iB_03691	TC004114	as expected	Aranda et al. 2007, Schoppmeier unpublished
decapentaplegic	iB_04497	TC008466	lethality of injected animals, differs from earlier experiments where strong embryonic defects, but no lethality were found	Van der Zee et al., 2006; Ober & Jockusch, 2006
dorsocross	iB_05219	TC012346	as expected, dorsal closure defect, appendage and abdominal defects	Panfilio unpublished, positive control in this study
EGF-Receptor	iB_00647	TC003986	as expected, oogenesis defects	Großmann, unpublished
folded gastrulation	iB_04203	TC006722	dorso-ventral patterning	Roth, unpublished
Frizzled-1	iB_02240	TC014055	early embryonic lethality, stronger defect than published	Beermann et al. 2011
glass-bottom-boat	iB_05543	TC014017	as expected, lipid homeostasis and sterility	Namigai & Suzuki 2012, Trauner unpublished
hairy	iB_05339	TC012851	as expected	Aranda et al. 2008
hunchback	iB_05451	TC013553	as expected	Marques-Souza et al. 2008
knirps	iB_03553	TC003413	early embryonic lethality, stronger defect than published	Cerny et al. 2008
lame-duck	iB_06061	TC030749	as expected, muscle defects	Frasch, unpublished
lim1	iB_05727	TC014939	early embryonic lethality, stronger defect than published	Posnien et al. 2011
methoprene-tolerant	iB_03648	TC003908	as expected, subtle leg defects	this study
mirror/irx	iB_03595	TC003634	as expected, oogenesis defects	Schoppmeier, unpublished
odd-skipped	iB_04013	TC005785	as expected	Choe et al. 2006
org-1	iB_05796	TC015327	as expected, muscle defects	Frasch, unpublished
patched	iB_03831	TC004745	as expected	Farzana et al. 2008

pelle	iB_02469	TC015365	as expected, strong embryonic defects	Roth, unpublished
porcupine	iB_03822	TC004714	as expected	Bolognesi et al. 2009
pumilio	iB_03898	TC005073	as expected	Schmitt-Engel et al. 2012
saxophone	iB_02534	TC015948	as expected, reduced fecundity	Roth, unpublished
sex-combs-reduced	iB_00186	TC000917	as expected, slightly stronger than published	Curtis et al. 2001; positive control in this study
sloppy-paired	iB_04421	TC008062	as expected	Choe et al. 2006, Choe et al. 2007
TGF-alpha	iB_03555	TC003429	as expected	Lynch et al. 2010; Roth unpublished
tolloid	iB_01822	TC011197	as expected	Fonseca et al. 2010
Torso	iB_04720	TC009906	as expected	Schoppmeier et al. 2005
torso-like	iB_04423	TC008090	as expected	Schoppmeier et al. 2005
twisted-gastrulation	iB_00592	TC003620	as expected	Fonseca et al. 2010
wingless	iB_05552	TC014084	as expected	Bolognesi et al. 2008
wnt-less	iB_00832	TC005345	as expected	Bolognesi et al. 2008
yb	iB_02707	TC000053	as expected	positive control in this study, Klingler unpublished
zfh2	iB_03646	TC003891	lethality of injected animals, differs from earlier experiments where defects of leg development, but no lethality were found	Prpic-Schäper, unpublished

Larval injection screen

**gene**	**iB-#**	**TC-#**	**screening result**	**reference**
achaete-scute	iB_04489	TC008433	as expected, lethality	positive control in this study
bric-a-brac	iB_03591	TC003621	just lethality, published subtle appendage phenotype was missed	Angelini et al. 2009, 2012
broad-complex	iB_03960	TC005474	as expected, slightly stronger	Konopova et al. 2008; Konopova, personal communication
cactus	iB_00322	TC002003	as expected	Fonseca et al. 2008; Roth unpublished
Delta	iB_03691	TC004114	larval lethality, stronger than published	Angelini et al. 2012a+b
decapentaplegic	iB_04497	TC008466	larval lethality, stronger than published	Knorr et al. 2009
ebony	iB_05139	TC011976	as expected	Park et al. 2005

EGF-receptor	iB_00647	TC003986	as expected, lethality	Großmann, unpublished
empty-spiracles	iB_05098	TC011763	wing and labrum defects, pupal lethality, differs from experiments in this study, where some lethality, but no morphological defect was found	positive control in this study
homothorax	iB_04526	TC008629	prepupal lethality, stronger than published	Angelini et al. 2012a+b
knickkopf	iB_04889	TC010653	as expected	Chaudhari et al. 2011
knirps	iB_03553	TC003413	prepupal lethality, differs from experiments in this study, where no phenotype was found, but earlier experiments showed similar defects	positive control in this study, Schmitt-Engel, unpublished
laccase 2	iB_01701	TC010489	larval lethality, stronger than published	Arakane et al. 2005
lim1	iB_05727	TC014939	as expected, slightly stronger	Angelini et al. 2012b
matrix metalloproteinase 1	iB_02266	TC014266	as expected	Knorr et al. 2009
mef2	iB_04920	TC010850	as expected, lethality	Frasch, unpublished
methoprene-tolerant	iB_03648	TC003908	larval lethality, technical artifact	Konopova et al. 2007; positive control in this study
odd-skipped	iB_04013	TC005785	just labial misorientation, weaker defect than published	Angelini et al. 2012
pumilio	iB_03898	TC005073	larval lethality, technical artifact	Schmitt-Engel, unpublished
serrate	iB_04764	TC010113	as expected, slightly stronger	Angelini et al. 2009
sex-combs-reduced	iB_00186	TC000917	as expected	Tomoyasu et al. 2005
sister-of-odd-and-bowl	iB_04014	TC005788	as expected, slightly stronger	Angelini et al. 2009, 2012a,b
Sp8	iB_05083	TC011697	as expected	Beermann et al. 2004
TGF-alpha	iB_03555	TC003429	as expected, morphological defects in pupae	positive control in this study
vestigal	iB_04931	TC010897	as expected	positive control in this study
wingless	iB_05552	TC014084	as expected, morphological defects in pupae and adults	positive control in this study
wnt-less	iB_00832	TC005345	as expected, morphological defects in pupae	positive control in this study
yb	iB_02707	TC000053	as expected, ovariogenesis defects	positive control in this study, Klingler unpublished

Supplementary Table 3 | Definition of phenotypic categories used in large scale comparison

Phenotype	Criteria
**no phenotype**	not in one of the other categories
**lethal after larval injection**	> 80 % of the injected animals died within 22 dpi
**parental lethal after pupal injection**	> 80 % of the injected animals died within 11 dpi
**embryonic lethal**	> 50 animals in cuticle preparation of clutch 9 dpi, or > 20 animals in cuticle preparation of clutch 9 dpi without any hatched animals, or > 20 animals in fresh preparation of clutch 11 dpi with > 50% embryos died before cuticle secretion/did not develop
**morphological defects of larval cuticle**	> 50 animals in cuticle preparation of clutch 9 dpi, while > 70 % hatched, preparation with > 50% larvae showing morphological defects but clearly visible polarity and at least 2 distinguishable tagmata; or > 50 animals in cuticle preparation of clutch 9 dpi, while 10 - 70 % hatched, preparation with > 30 % larvae showing morphological defects but clearly visible polarity and at least 2 distinguishable tagmata; or > 20 animals in cuticle preparation of clutch 9 dpi while no animals hatched, preparation with > 30 % larvae showing morphological defects but clearly visible polarity and at least 2 distinguishable tagmata
**strong defects of larval cuticle**	> 50 animals in cuticle preparation of clutch 9 dpi, while > 70 % hatched, preparation with > 50 % larvae without clear tagmatic division or polarity; or > 50 animals in cuticle preparation of clutch 9 dpi, while 10 - 70 % hatched, preparation with > 30 % larvae without clear tagmatic division or polarity; or > 20 animals in cuticle preparation of clutch 9 dpi while no animals hatched, preparation with > 30 % larvae without clear tagmatic division or polarity
**embryonic lethal before cuticle secretion**	> 50 animals in cuticle preparation of clutch 9 dpi, preparation with > 50 % egg shells without larval cuticle or >30 % egg shells without larval cuticle in combination with morphological defects (see above); or > 20 animals in cuticle preparation of clutch 9 dpi while no animals hatched, preparation with> 50 % egg shells without larval cuticle or >30 % egg shells without larval cuticle in combination with morphological defects (see above); or > 20 animals in fresh preparation of clutch 11 dpi with > 50% embryos died before cuticle secretion/did not develop
**defects in metamorphosis control**	> 3 injected animals died during development from prepupal to adult stage
**defects of adult structures**	> 2 injected animals with morphological defects of pupal or adult structures (except ovary defects)
**ovary defects**	> 50 % of dissected animals with reduced egg production show morphological defects of ovaries and no reduced fat body 22 dpi or 13 dpi
**morphological defects of pupae or adults**	> 2 injected animals with morphological defects of external pupal or adult structures

**morphological defects of pupal or adult legs**	> 2 injected animals with morphological defects of pupal or adult legs
**morphological defects of larval legs**	> 50 animals in cuticle preparation of clutch 9 dpi, while > 70 % hatched, preparation with > 50% larvae showing leg defects; or > 50 animals in cuticle preparation of clutch 9 dpi, while 10 - 70 % hatched, preparation with > 30 % larvae showing leg defects; or > 20 animals in cuticle preparation of clutch 9 dpi while no animals hatched, preparation with > 30 % larvae showing leg defects
**defects of adult thoracic musculature**	> 2 injected animals with defects of the developing dorsal thoracic musculature (marked at pupal stage by d17 enhancer trap)
**defects of larval musculature**	> 20 animals in fresh preparation of clutch 11 dpi with > 30% embryos/larvae showing defects of musculature (marked by pig19 enhancer trap)
**odoriferous glands defects**	> 1 injected animal showed aberrations of abdominal or thoracic odoriferous glands
**blistered wings phenotype**	> 4 injected animals show a total or partial separation of dorsal and ventral wing surfaces

Note: We developed criteria for selecting datasets with high likelihood of reproducibility for our dataset wide analysis (shown in Figs. 2 and 3). For each phenotype class, we defined specific cut-off values, which were based on our experience gained during the reproduction experiments (e.g. reproducibility, see Figure 1d and Supplementary Figure 1). The main criterion was the penetrance of a phenotype (>50% for most embryonic phenotypes; >2 animals for most phenotypes observed in the injected animals; >80% penetrance for lethality). This main criterion was complemented with other criteria wherever our experience indicated that we would miss a significant number of phenotypes when only applying penetrance.

Supplementary Table 4 | *Tribolium* wing-blister genes tested in *Drosophila*

iB#	*D.m. ortholog*	Driver:	Bbg-GAL4	Bx-GAL4
			**Description of wing phenotype**	
Annotations associated with wing blister in Drosophila				
iB_00385	*Ilk (integrin linked kinase)*		blister	crumpled
iB_05522	*Asx (additional sex combs)*		WT	blister, curly
iB_05688	*wb (wing blister)*		blister	blister
iB_00014	*parvin*		blister	blister
Annotations associated with cytoskeleton in Drosophila				
iB_05272	*Mob4*		WT	unable to eclose
iB_00101	*TBCB*		WT	blister
iB_02017	*CG32138*		WT	vein defect
Annotations associated with cell adhesion in Drosophila				
iB_01705	*LanB2 (Laminin B2)*		WT	blister
iB_01221	*Cka (Connector of kinase to AP-1)*		crumpled	wings reduced, blisters unsure
iB_01762	*Pak (PAK-kinase)*	line 1	crumpled	crumpled
		line 2	WT	WT
		line 3	WT	curly, crumpled
iB_00557	*Lar (Leukocyte-antigen-related-like)*		crumpled	curly, shape, low hatchrate
iB_00666	*Sra-1 (specifically Rac1-associated protein 1)*		WT	crumpled
Annotations without cell adhesion or cytoskeleton in Drosophila				
iB_01726	*CG11526*		crumpled, mis-shapen, low hatchrate	wings reduced, blisters unsure
iB_02548	*CG5734*		WT	WT
iB_00300	*plexA (plexin A)*		WT	potential blisters
iB_00499	*SRPK*	line 1	vein defects	vein defects
		line 2	WT	blister
iB_00907	*Eip71CD (Ecdysone-induced protein 28/29 kD)*		WT	curly
iB_00845	*Lrt (Leucine-rich tendon-specific protein)*		WT	WT
iB_04887	*CG8078*		WT	blister, low hatchrate
PS integrins as positive controls (not included in iBeetle screen)				
-	*mew (multiple edematous wings)*		WT	blister
-	*mys (myospheroid)*		unable to hatch	blister, low hatchrate
-	*if (inflated)*		WT	WT

Supplementary Table 5 | *Drosophila* wing blister phenotypes in *Tribolium*

iB-number	name of *Drosophila* ortholog	iBeetle phenotype (Injection of L5/6)	Rescreen phenotype (Injection of L7)
iB_01796	*papillote*	larval lethal	larval lethal
iB_01467	*kopupu/shortstop/kakapo*	larval lethal	larval lethal
iB_03871	*blistered*	larval lethal	blistered wings
iB_05141	*dumpy*	larval lethal	larval lethal
iB_04642	*piopio*	larval lethal	larval lethal
iB_05522	*xenicid/additional sex combs*	blistered wings, deformed genital lobe	-
iB_01537	*rhea/talin*	larval lethal	blistered wings
iB_03691	*delta*	larval lethal	pupal lethality without blistering

Note: The database was screened for genes known to elicit a wing blister phenotype in Drosophila (based on Prout et al. 1997 and Walsh and Brown 1998 [5,6]). In seven out of eight cases, death prior to wing formation had prevented detection of the blister phenotype ("iBeetle phenotype"). We reasoned that injection at the last larval stage (L7) would reduce lethality and, hence, allow the detection of potential wing blister phenotypes. Indeed, two more genes showed the wing blister phenotype after later injection ("Rescreen phenotype"). In total, three out of eight genes (38%) would have been detected in a screen using systemic RNAi in the last larval stage.

Supplementary Table 6 |Workflow of the pupal and larval injection screens

Days after injection	Processing step	Phenotypic aspect screened
**Workflow pupal injection screen**		
**d0**	Injection (10 female pupae)	
**d3**	Inspection of adults	Metamorphosis control
**d3-9**	1st egg collection	
**d9**	Inspection of adults	Lethality of injected animals
**d9-11**	2nd egg collection	
**d11**	Assessment number of eggs in 2nd collection	Egg productivity
**d13**	L1 cuticle preparation<br>(based on 1st egg collection)<br>Dissection of ovaries*	Ovary morphology*
**d14**	Muscle preparation<br>(based on 2nd egg collection)	L1 somatic muscles
**d27-32**		L1 cuticle morphology#

**Workflow larval injection screen**		
**d0**	Injection (10 female larvae)	
**d11**	Inspection of pupae	Lethality of injected animals<br>Metamorphosis control<br>Pupal morphology<br>Pupal muscles
**d16/18**	Inspection of adults	Metamorphosis control<br>Adult morphology<br>Melanization
**d20-23**	Egg collection	
**d21-25**	Assessment number of eggs<br>Dissection ovaries*	Egg productivity<br>Ovary morphology*
**d38-41**	Analysis stink glands#	Altered or missing stink glands

*If number of eggs was reduced in second egg collection #Exact timing not critical

Supplementary Table 7 | Sequences of the dsRNA fragments used for the analysis of selected genes

iB-number	iB-fragment	Non-overlapping fragment
iB_05549	AGCACCAGACCCAGAACAACAACAACAACAATACCAAATCGCAAAAC<br>ATGGAGCGGGTCAAACGGCCCATGAACGCCTTCATGGTGTGGTCGCG<br>GGGCCAGCGGCGGAAAATGGCCCAGGAAAACCCCAAAATGCACAATT<br>CGGAAATCTCGAAGCGGCTGGGGGCCGAGTGGAAGCTGTTGAGTGA<br>GGCCGAGAAGCGGCCCTTCATCGACGAGGCCAAGCGGCTGAGGGCC<br>GTGCACATGAAGGAACACCCGGATTACAAGTACCGGCCTAGGAGGAA<br>GACCAAGACGCTCCTGAAGAAGGATAAATATCCCTTGGGAGCGTCCA<br>GCTTGATCCCGACTAGTGACCCGACGCGGACGGCGCCTTCGGCGGTC<br>CAACAGGTGTCCAGCCGGGACATGTACCAGATGCCCAATGGGTACAT<br>GCCCAACGGGTACATGATGCACGACCCCGGGGCCTACCAGCAGCAGT<br>ACACCGGGTCCAACTACGGCCGCTACGACATGTCGCAAATGCAGTACA<br>TGAACGGTTACGGTTACGG	ACCAAGACGCTCCTGAAGAAGGATAAATATCCCTTGGGAGCGTCCA<br>GCTTGATCCCGACTAGTGACCCGACGCGGACGGCGCCTTCGGCGGT<br>CCAACAGGTGTCCAGCCGGGACATGTACCAGATGCCCAATGGGTAC<br>ATGCCCAACGGGTACATGATGCACGACCCCGGGGCCTACCAGCAGC<br>AGTACACCGGGTCCAACTACGGCCGCTACGACATGTCGCAAATGCA<br>GTACATGAACGGTTACGGTTACGGGGCCACCGTGCCCCAGAGTGCA<br>GGCTCCCCCTACGGAATGCAACAGACGTCGTCGCATAGCCCCTCTG<br>GGTCCAGTATAAAATCGGAGCCGGTTTCTCCGAGTTCGGGGCTGCA<br>CACACCGACGCCGGGCGTCAAGCGGGAGTACGGCCAACAGCAGCA<br>GCCCCAGGGGGACCTGCGACAGATGATCTCCATGTACCTGCCCAC
iB_05264	AAGGGCTACTGGGGTTTCACCCGGCGATTACAAAGATTTCAAACCAAA<br>CAATCCCGATATTAAAGTCGAAGACGGCACTCTTACTATTAACAATATC<br>CAGAAAACAAACGAGGGTTATTATTTATGTGAGGCTGTCAATGGGATT<br>GGATCAGGATTATCTGCAGTTATTCAAATCAGTGTTCAAGCTCCCCCAC<br>AGTTTGATATTAAACTCAGGAACCAAACCTCCCGCCGTGGAGACCCTG<br>CCGTCCTCCAATGTGAGGCCAAAGGCGAAAAACCGATTGGTATTTTAT<br>GGAATATCAACAATAAGCGTTTGGAACCAAAAGGCGACAATAGATAC<br>ACGATCCGGGAGGAGATCCTCGCCAATGGTGTTCTTTCCGGCCTCAGT<br>ATCAAACGCACAGAACGCTCCGACTCCGCTCTCTTTACTTGTGTAGCTA<br>CCAACGCCTTCGGCAGTGACGATACCAGCATTAACATGATTGTGCAAG<br>AAGTACCAGAGGTACCATACGGGCTGAA	AAGGGCTACTGGGGTTTCACCCGGCGATTACAAAGATTTCAAACCA<br>AACAATCCCGATATTAAAGTCGAAGACGGCACTCTTACTATTAACAA<br>TATCCAGAAAACAAACGAGGGTTATTATTTATGTGAGGCTGTCAAT<br>GGGATTGGATCAGGATTATCTGCAGTTATTCAAATCAGTGTTCAAG<br>CTCCCCCACAGTTTGATATTAAACTCAGGAACCAAACCTCCCGCCGT<br>GGAGACCCTGCCGTCCTCCAATGTGAGGCCAAAGGCGAAAAACCG<br>ATTGGTATTTTATGGAATATCAACAATAAGCGTTTGGAACCAAAAG<br>GCGACAATAGATACACGATCCGGGAGGAGATCCTCGCCAATGGTG<br>TTCTTTCCGGCCTCAGTATCAAACGCACAGAACGCTCCGACTCCGCT<br>CTCTTTACTTGTGTAGCTACCAACGCCTTCGGCAGTGACGATACCAG<br>CATTAACATGATTGTGCAAGAAGTACCAGAGGTACCATACGGGCTG<br>AA
iB_04564	CGTCTACAGCATCGACCAGATTTTGGGAGTTAACAGCTCGTCCACCTCT<br>GCGAAGAGCATCGAAGGCGAGTCGGATTCCAAAGTCGATTCGGTCAG<br>TGATAGCGAAATGGTTGAGGAAAGCATCGAAGATTTGAACGACACCA<br>GACCGAGAAAAATCCGCAGATCCCGAACGACTTTCACCACCTATCAGC<br>TGCACCAACTGGAGCGAGCTTTCGAAAAAACCCAATACCCCGACGTTT<br>TCACAAGAGAGGAATTGGCTATGCGGCTTGATTTGAGCGAAGCGCGA<br>GTCCAGGTATGGTTCCAAAACCGTCGCGCCAAGTGGAGAAAGCGTGA<br>AAAGG	CGCTTTCCTGTCGCAATACGTGCTCGCAGGCGGCGTCCCCCAACTGA<br>ATCTCCTAAATAGTGGGATTCCGGACGAACGGTCCCGGAGTACGAG<br>TCCGGAAACTCCCAGTCCGTCGCAGTTGTCGCCCTCAGCGCTGGAA<br>GCTTTGAGGTTACGGACACAAGAAATTTTAGTGCCGAGTTCTTCGCC<br>GCAGAAACTGCACGCGAAGTCGTAAATAGATAATATCTAGTGGACA<br>TGTAGTTGTATTGTTAGGGATGTAGGAGATTATTGGCGCTTGATAT<br>GTGGGGGAAATAGACTGAATAGCTGTACCAAGTTTTCGATTTAGTT<br>GTTATTTTTTTTTCATTCTTATGATAGGGAGAATTATTTTTGTATTAA<br>ATTTATTTACAACACTAGTTGCACTAGTTAGTTTAGCGCCAATTTTGT<br>AAATAATTTTGTACATAAAGTGCGTATTTATTTATTGTAGATAAAAA<br>ATCAAGAGGTTTGTTTAAAACATAATAGCATTACAGATAATTATTTT<br>GTGGGGGAAATTAACAATAAGTTGTACCTAATACCTAGTTACGATC<br>GATATTAAACGAAACAATAATTAAAACGGCAGGTGTTTTACTTGTCT<br>TGGT
iB_00289	TCAGAGTGCGAAAATGCTGATGCCCAGTGGACTTCCGGCCAACGAGG<br>CCGATGGTCTTGGACTCGGAGGCCTGGGCACGAACACAAACACCAAC<br>AACGGAGTGCAGCATAAGGACACCACATGGACCAAACTGTTCGTGGG<br>TGGACTGCCGTATCACACCACGGATCAGTCGCTAAGAGAACATTTTTC<br>GGTTTATGGGGAAATCGAAGAAGCGGTGGTCATCACTGATAGACAAA<br>CGGGGAAAAGTCGAGGATATGGATTCGTAATTATGGGAGATAAGTCT<br>TCATCAGATAGAGCATGTAAAGACGCTAACCCCATTATTGACGGGCGA<br>AAAGCGAATGTGAATTTAGCGATTCTGGGAGCGAAACCGAGAGGAAA<br>TGCGCAAACGGGTTTCCCGTTTCAAGGAATCCGGGCTGGTTATCCTGC<br>ACTTCTTCCGGGCCAGTACGGAATGCCTCCTGGTTATGTGTACCAATCT<br>CCATACCTAACTGCTGCTGCTCCTGGAAGTCT	CGCTAACCCCATTATTGACGGGCGAAAAGCGAATGTGAATTTAGCG<br>ATTCTGGGAGCGAAACCGAGAGGAAATGCGCAAACGGGTTTCCCG<br>TTTCAAGGAATCCGGGCTGGTTATCCTGCACTTCTTCCGGGCCAGTA<br>CGGAATGCCTCCTGGTTATGTGTACCAATCTCCATACCTAACTGCTG<br>CTGCTCCTGGAAGTCTT
iB_00555	GCTGATTGC ATTCTCCAA CAAGCGGTA GTCGGTGCC GGGGCCAAT<br>CAGTTGGTT TTGAGTTAC TTGAGACAC TCCTTGAGC GCCCAGTTA<br>GTCTCACAT GCGGCTGTG CTTCAACGC CTCAGCAAA TACAACCAA<br>CTCAGTAAA GTCCACTGC GTGTTTAGC CTACTTGAG TTCCTCGAA<br>GGCATGCTT CCGGGTGTC ACTTGTTGT GGCAAACCG GAAGAAACC<br>GTCCTGGCC ACTGCAATT TTATCAATT GCTTGTTGG CTCCTGACT<br>ATTTTATTG CAATGCAAA GGCACCGCT CATTTGACT CAGAAAGCC<br>TCGTTCTTG CTCACCACG CTCATGAAT GACGATTTT TACGTCTCC<br>ATGATGTGT CTAGCGAGG TACAGCGAT CCTGAATTG TTTACTGAG<br>ACCAACCGC AAATGTATT GAGTTGAGG GCGTCGTTG TCCGATTCT<br>GACGAGTTA TCTAAATGC GTTAAAAAA CTGGAAAAC ATCGATGTT<br>AACATTTTG AGCCTCCCG ATCAATAG	TGCAGAGAGGTGTGGATCTGGCTTTGGCGGCGTTCCATGTCGATAT<br>CAGGGCCTGCACTTTGGAGCTGCTCTCGCATGTTTTGCCCCAAATGT<br>TGTATAACGATCTCCAAGCGGACTCGCTCATGGAGCCGCATTTGATC<br>GCATTGGCTTACTTGACCAGTTATTGCGTGTACACGGCGTTTGATGC<br>GTTTTCGGAAGAACCGGAAGAACCGATGGCTAAAGTTGCGCGGCTT<br>AATGAGGGTGAGGATGGCACGCTGATTGAGCAACTAATTTCGACTC<br>TCAGACAACTGATGACGATTTTTGAGGATGGGATACAAGAGGGTTA<br>CATCACTCAACAGACTTATTTCGCGTTTTATTTGATTAAAAGTCTGGT<br>TGAGGTGAAAGTGAGCACGGCGAGTGCTCTCTTGGCTGCGATTCCG<br>CCGGCTCTTGTGACAGACTTGCTTCGGACTCTGCCTGAACTTTTTAC<br>TTACCCGATTTTGTTGCATCTGCACGATGTGTTTAATACTCACGGTC<br>GGAATAACATGGCAAAGGACTTGTGTATATTGCGAAATTACCACTT<br>GAGGAACGTTTCG
iB_04537	TCATGCAGAAACGGGGTACTCCGATCAATCGACTGCCCATCATGGCCA<br>AATCCGTTCTCGATTTATACGAATTGTACAATTTAGTCATCGCGAGGG<br>GTGGACTTGTTGATGTCATCAATAAGAAACTCTGGCAGGAAATTATCA<br>AAGGGCTGCATCTACCGTCGTCGATAACGTCGGCAGCTTTCACACTCA<br>GGACGCAATACATGAAATACCTGTACCCTTACGAATGCGAGAAACGCC<br>GCCTCAGCACACCGGCCGAGCTCCAGGCGGCCATCGACGGCAACCGT	CGCATGATGGAATACGTCAAGCTCCTCAACAAGGAAATCCGCAGCT<br>CGGCGGCTACTCCGCCACGACAAGGGGACGTGTCGCCCCCTAACGC<br>CACCTCGCCACTCAACCAGCTGGAGCTGTCGCGGATAACGCTCTGG<br>AATATGTACAATAATAATCAACCCCCCGTCGAGCCGCAAAAGGAAG<br>CGTTGAATCTCTCCGACCCGACGCAGCCTTCGGTGAAACGGGAGCC<br>GGAGCATAGAGATTCGCCACCGCCCCCGAAGAAGTTTTCCAAAGAT

	CGCGAAGGCCGCCGCAGCAGCTACGGCCAGTACGACTCCATGCAGCG<br>CTCCCCCAACCCTTCGCAGATGTCTCCTTTGTCCCTCGTC	GATGATGAACAGAAGATCTCGCCC
iB_02517	TCACTCAAAGCCACAGATGCGGTTTTAGTAAAATTGGGGCCCAAGGG<br>CGAAATTTCAAATGAGACACTTGTTCACGTCGATTTGGTGCAACGTGG<br>GGATGTTTTAAAAGTGGTACCGGGGGCCAAAGTACCAGTCGACGGCA<br>AAGTTTTACAGGGCCAATCAATGTGCGACGAGAGCCTCATAACTGGG<br>GAAAGCATGCCGGTACCGAAGAAAATCACAAGTAGTGTGATTGGTGG<br>ATCAATCAATCAGCACGGCCTACTTATAATCGAAGCCACACATACAGG<br>AGAGGCAACAACCCTATCACAAATTGTCAAATTGGTCGAAGAAGCACA<br>AACGTCAAAAGCACCCATCCAACAATTGGCCGATAAAATCGCTGGTTA<br>TTTCGTCCCAACTGTCGTCTTCCTGTCACTTTTGACACTTATTGTCTGGT<br>CTATTATCGGCTCAATCGATATAAACGCACTTCCGGTGAC	CCCCACAGGCGACTACACCTCCGACCAACCAACAATAATCACAGTG<br>TCAGAAGATGACACGATTAAAATCACCGTCTTGGGCATGACGTGCC<br>AAAGCTGTGTCAAAAACATCGAAGAGACCCTGAGTCGTAAACCCGG<br>CATTTACAACATCAAAGTCAGCCTTCAGGAAAAAGCCGCTCTAGTCC<br>ATTATGACACACGCCAACTGACA
iB_04887	GGTGGCAAAGACTCCACAGTTCTGGCGTACGTCATGAAGCTACTTAAC<br>GAAAAGTACGATTATAAGCTGGACTTAGTGCTTTTGTCCATTGATGAG<br>GGTATTACAGGGTACAGGGACGATAGTTTAGATACTGTGAAACAAAA<br>TCGGGACGATTACGGAATGCCTTTGAAAATAATGTCTTATAAGGATTT<br>GTACGGTTGGACAATGGACGAAATTGTGGCTGAGATTGGGAGGAAA<br>AATAACTGTACTTTTTGTGGCGTTTTCAGACGCCAGGCTTTAGACAGA<br>GGGGCGGCTCTTCTAAATGTTGATTATTTAGCAACTGGACATAACGCT<br>GATGATATTGCAGAGACTGTCTTGATGAATATTTTAAGGGGCGATTTG<br>GCACGTCTCAGCCGTTGTACGTCCATTATCACGGACAGTGGTGACGGC<br>ATTCCACGCGTAAAACCCCTCAAATACACCTACGAGAAAGAAATCGTC<br>ATGTACGCCT	TCTCCACTGAGTGTGTCTTCGCCCCCAATGCGTACAGAGGCCATGCC<br>CGGGTGTTGCTCAAAGATTTGGAAAAAATAGACCCTGCTGTTATAA<br>TGAATATTATCCAGTCGGGGGAATCCCTCAAAATCAACGAAAATGC<br>CAATATGCCAACTTTGCAGAAATGCACAAGGTGCGGATATGTGTCG<br>TCGCAGGACGTGTGCAAAGCTTGCGTCCTCTTGGAGGGACTCAATA<br>AAGGATTACCGAAACTAGGAATAGGGAAGTCGAGTAAAGTGAAAC<br>GACATTTACAAGAAAACAGTCCGTGTTGTAAAACGC
iB_05264	AAGGGCTACTGGGGTTTCACCCGGCGATTACAAAGATTTCAAACCAAA<br>CAATCCCGATATTAAAGTCGAAGACGGCACTCTTACTATTAACAATATC<br>CAGAAAACAAACGAGGGTTATTATTTATGTGAGGCTGTCAATGGGATT<br>GGATCAGGATTATCTGCAGTTATTCAAATCAGTGTTCAAGCTCCCCCAC<br>AGTTTGATATTAAACTCAGGAACCAAACCTCCCGCCGTGGAGACCCTG<br>CCGTCCTCCAATGTGAGGCCAAAGGCGAAAAACCGATTGGTATTTTAT<br>GGAATATCAACAATAAGCGTTTGGAACCAAAAGGCGACAATAGATAC<br>ACGATCCGGGAGGAGATCCTCGCCAATGGTGTTCTTTCCGGCCTCAGT<br>ATCAAACGCACAGAACGCTCCGACTCCGCTCTCTTTACTTGTGTAGCTA<br>CCAACGCCTTCGGCAGTGACGATACCAGCATTAACATGATTGTGCAAG<br>AAGTACCAGAGGTACCATACGGGCTGAA	GTTTTGGGTTACCGCAGCTACCACTATTGGAGAAGGGCAACCGTCG<br>AAGAAAGTTACAGTGTCTCCAAGCGCGAGCGTTCCAGCCAAAATCG<br>CCTCGTTTGACGATACCTTCACCACGACGTACAAGGAAGACGTGAC<br>TCTCCCTTGCCTCGCCGTTGGGTTGCCACCACCGGTCATCACATGGA<br>AAATCAAGGGGGTTCAGTTCACCACAAGCGACAAAATCAGGCAACA<br>ACCAGACGGGTCACTGTTTATTCGTGATGTCAGTCGGAATAACGCA<br>GGGGAGTACTCGTGTCACGTTGAGAATGACTATGGACAGGACTCG<br>GTGACTCACCAGTTGATTGTCAATGCTCCTCCACACGCACCACAAAT<br>TGCTCTCACTTCAACTACCACAAATTCGCTCACGTTTAAGTTAAAGCC<br>GCATGAGTCGGATGTTGAGCCGATCCATGGATACACTATTCACTAC<br>AAGCCAGAGTTTGGCGATTGGGAGACGGTCCAGATTGGACCAACT<br>GTCGAAAAGTACACTTTGGAGAAGTTGCTGTGTGG
iB_04564	CGTCTACAGCATCGACCAGATTTTGGGAGTTAACAGCTCGTCCACCTCT<br>GCGAAGAGCATCGAAGGCGAGTCGGATTCCAAAGTCGATTCGGTCAG<br>TGATAGCGAAATGGTTGAGGAAAGCATCGAAGATTTGAACGACACCA<br>GACCGAGAAAAATCCGCAGATCCCGAACGACTTTCACCACCTATCAGC<br>TGCACCAACTGGAGCGAGCTTTCGAAAAAACCCAATACCCCGACGTTT<br>TCACAAGAGAGGAATTGGCTATGCGGCTTGATTTGAGCGAAGCGCGA<br>GTCCAGGTATGGTTCCAAAACCGTCGCGCCAAGTGGAGAAAGCGTGA<br>AAAGG	CGCTTTCCTGTCGCAATACGTGCTCGCAGGCGGCGTCCCCCAACTGA<br>ATCTCCTAAATAGTGGGATTCCGGACGAACGGTCCCGGAGTACGAG<br>TCCGGAAACTCCCAGTCCGTCGCAGTTGTCGCCCTCAGCGCTGGAA<br>GCTTTGAGGTTACGGACACAAGAAATTTTAGTGCCGAGTTCTTCGCC<br>GCAGAAACTGCACGCGAAGTCGTAAATAGATAATATCTAGTGGACA<br>TGTAGTTGTATTGTTAGGGATGTAGGAGATTATTGGCGCTTGATAT<br>GTGGGGGAAATAGACTGAATAGCTGTACCAAGTTTTCGATTTAGTT<br>GTTATTTTTTTTTCATTCTTATGATAGGGAGAATTATTTTTGTATTAA<br>ATTTATTTACAACACTAGTTGCACTAGTTAGTTTAGCGCCAATTTTGT<br>AAATAATTTTGTACATAAAGTGCGTATTTATTTATTGTAGATAAAAA<br>ATCAAGAGGTTTGTTTAAAACATAATAGCATTACAGATAATTATTTT<br>GTGGGGGAAATTAACAATAAGTTGTACCTAATACCTAGTTACGATC<br>GATATTAAACGAAACAATAATTAAAACGGCAGGTGTTTTACTTGTCT<br>TGGT
iB_00289	TCAGAGTGCGAAAATGCTGATGCCCAGTGGACTTCCGGCCAACGAGG<br>CCGATGGTCTTGGACTCGGAGGCCTGGGCACGAACACAAACACCAAC<br>AACGGAGTGCAGCATAAGGACACCACATGGACCAAACTGTTCGTGGG<br>TGGACTGCCGTATCACACCACGGATCAGTCGCTAAGAGAACATTTTTC<br>GGTTTATGGGGAAATCGAAGAAGCGGTGGTCATCACTGATAGACAAA<br>CGGGGAAAAGTCGAGGATATGGATTCGTAATTATGGGAGATAAGTCT<br>TCATCAGATAGAGCATGTAAAGACGCTAACCCCATTATTGACGGGCGA<br>AAAGCGAATGTGAATTTAGCGATTCTGGGAGCGAAACCGAGAGGAAA<br>TGCGCAAACGGGTTTCCCGTTTCAAGGAATCCGGGCTGGTTATCCTGC<br>ACTTCTTCCGGGCCAGTACGGAATGCCTCCTGGTTATGTGTACCAATCT<br>CCATACCTAACTGCTGCTGCTCCTGGAAGTCT	GGAGCACCTTCACCAAAGAGTACATAAACAACGTGGTTAACCCCCT<br>CGGAAACCGGCCCACCAGACCAACGGAAAACCACACTACCACCTCC<br>TCGGAGCGTTCCGCAGCTTCTGATCCTTCTGAAGATCAGAGTGCGA<br>AAATGCTGATGCCCAGTGGACTTCCGGCCAACGAGGCCGATGGTCT<br>TGGACTCGGAGGCCTGGGCACGAACACAAACACCAACAACGGAGT<br>GCAGCATAAGGACACCACATGGACCAAACTGTTCGTGGGTGGACT<br>GCCGTATCACACCACGGATCAGCGCTAACCCCATTATTGACGGGCG<br>AAAAGCGAATGTGAATTTAGCGATTCTGGGAGCGAAACCGAGAGG<br>AAATGCGCAAACGGGTTTCCCGTTTCAAGGAATCCGGGCTGGTTAT<br>CCTGCACTTCTTCCGGGCCAGTACGGAATGCCTCCTGGTTATGTGTA<br>CCAATCTCCATACCTAACTGCTGCTGCTCCTGGAAGTCTT
iB_04887	GGTGGCAAAGACTCCACAGTTCTGGCGTACGTCATGAAGCTACTTAAC<br>GAAAAGTACGATTATAAGCTGGACTTAGTGCTTTTGTCCATTGATGAG<br>GGTATTACAGGGTACAGGGACGATAGTTTAGATACTGTGAAACAAAA<br>TCGGGACGATTACGGAATGCCTTTGAAAATAATGTCTTATAAGGATTT<br>GTACGGTTGGACAATGGACGAAATTGTGGCTGAGATTGGGAGGAAA<br>AATAACTGTACTTTTTGTGGCGTTTTCAGACGCCAGGCTTTAGACAGA<br>GGGGCGGCTCTTCTAAATGTTGATTATTTAGCAACTGGACATAACGCT<br>GATGATATTGCAGAGACTGTCTTGATGAATATTTTAAGGGGCGATTTG<br>GCACGTCTCAGCCGTTGTACGTCCATTATCACGGACAGTGGTGACGGC<br>ATTCCACGCGTAAAACCCCTCAAATACACCTACGAGAAAGAAATCGTC<br>ATGTACGCCT	TCTCCACTGAGTGTGTCTTCGCCCCCAATGCGTACAGAGGCCATGCC<br>CGGGTGTTGCTCAAAGATTTGGAAAAAATAGACCCTGCTGTTATAA<br>TGAATATTATCCAGTCGGGGGAATCCCTCAAAATCAACGAAAATGC<br>CAATATGCCAACTTTGCAGAAATGCACAAGGTGCGGATATGTGTCG<br>TCGCAGGACGTGTGCAAAGCTTGCGTCCTCTTGGAGGGACTCAATA<br>AAGGATTACCGAAACTAGGAATAGGGAAGTCGAGTAAAGTGAAAC<br>GACATTTACAAGAAAACAGTCCGTGTTGTAAAACGC

Supplementary Table 8 | Comparison of number of lethal genes

Drosophila				
	**Absolute numbers**	**% related to …**		**Reference**
**Protein coding genes**	13,918	…all genes:		FlyBase; Release 6.02
**Lethal loci**	5,000	35.9%		Nüsslein-Volhard 1994
**Sterile loci**	1,000	7.2%		Nüsslein-Volhard 1994
**Essential genes (sum lethal plus sterile)**	6,000	43.1%		Nüsslein-Volhard 1994
		... lethal genes:		
**Embryonic lethal genes**	1,000	20.0%		Mullins et al. 1994
		... embryonic lethal genes:		
**Embryonic lethal without cuticle phenotype**		85.0%		Mullins et al. 1994
**…with phenotype**		15.0%		

Tribolium				
	**Numbers found in the screen**	**Numbers corrected for false positive rate of 26%**	**% of corrected numbers related to …**	**Reference**
**All analyzed genes**	3,400		… all analyzed genes	
**Lethal genes**	1,686	1,248	36.7%	Fig. 2b, heading
**Non-lethal genes with phenotype**	229	169	5%	Fig. 2a
			… all lethal genes	
**Embryonic lethal genes**	969	717	57.5%	
			… embryonic lethal genes	
**No cuticle defect**	186	138	19.2%	
**With cuticle defect & empty egg**	783	579	80.8%	

Supplementary Note 1

Detailed information on positive controls

In order to assess reliability and sensitivity of our screen, and to test the alertness of the screeners, we included 41 different positive controls. The screeners did not know which gene was used as positive control in a given repetition, whether this control had a phenotype in the pupal or the larval screen, and due to the high number of different controls, a screener would encounter most controls only once during the screen. However, for technical reasons in the production pipeline, it was not possible to hide the position of positive controls within a repetition. Hence, with some effort, a screener could identify the position of the positive control and scrutinize it more carefully (see details below). As an additional way assessing reliability and sensitivity of the screen, we searched for datasets that by chance had targeted genes with known phenotypes. The identities of these genes were neither known to the screeners nor the PIs such that these datasets represent double blind positive controls (see details below). We observed similar recognition rates as in the added positive controls (see Fig. 1a and b).

It turned out that in our procedure, the phenotypes of some positive controls were reproducibly different from the published phenotypes and novel phenotypic aspects were detected (e.g. due to different dsRNA concentration or different injection stage and timing). Therefore, the entire iBeetle procedure was performed with all positive controls in order to define the phenotypic aspects that had to be recognized in our screen.

In 370 cases (93.2%, n=397) the phenotype was entirely (83.3%) or partially (9.8%) recognized (dark and light green; Supplementary Figure 1). Controls were regarded as "partially recognized" when the majority (but not all) of the aspects of a phenotype were correctly annotated.

Most of the 39 cases that were only partially recognized were derived from five positive control genes. These genes had originally been chosen for controlling the pupal injection screen but turned out to have phenotypes in the larval screen as well (*Tc-vestigial, Tc-hedgehog, Tc-wnt-less/evi* and *Tc-TGF-alpha*; see controls marked with a diamond in Supplementary Figure 1). We scored them as "partially recognized" when at least three out of four aspects were recognized. Four additional cases of partially recognized controls stem from *Tc-metoprene tolerant (Tc-met),* where the lethality of the injected animals was properly annotated but the necrotic head was missed.
With respect to "technical lethality", in 11 cases (2.8%) the premature death of the injected animals prevented detection of the phenotype (Supplementary Figure 1, light blue). 16 cases were true false negatives (4%; Supplementary Figure 1, dark blue). 62.5% of those cases derived from the analysis of only three positive control genes that elicited subtle morphological defects in the adults, like slightly deformed cuticular structures at the ventral midline of adult beetles after larval *Tc-orthodenticle* RNAi (missed five times), or minor head or leg defects after pupal injections of *Tc-methoprene-tolerant* or *Tc-aristaless* (together five cases).

We analyzed 65 datasets that by chance had targeted genes with previously described phenotypes (

Supplementary Table 2). Hence, this set represents completely blind positive controls. 51 of those (78.5%) were recognized with the previously published phenotype.

In eleven cases (16.9%), we found a phenotype which was reproducibly different from the published one (i.e. reproduced in an independent repetition of the experiment following our screening procedure). Hence, the phenotype differed due to our specific experimental conditions, not due to experimental variation. The relevant parameters responsible for such differing phenotypes could be the different stage and timing of injection, the genetic background of the injected animals and the different dsRNA fragment used for the experiments.

We observed several cases where the RNAi phenotypes were reproducibly different from published data. One case is likely due to strain specific differences: The larval injection of two different fragments against *Tc-empty spiracles* (iB_05098 and a positive control fragment) reproducibly elicited different phenotypes in two different strains. In case of *Tc-odd-skipped* (iB_04013) the difference could be either strain specific or due to different dsRNA concentrations. Larval injection of the iB-fragment in L6 or L7 larvae did not lead to any phenotype in the screen, although defects of mouthparts, antennae and legs had been described previously [7–9]. However, in our hands, neither the iB-fragment nor a non-overlapping fragment, either in the screening strain or in another genetic background, reproduced the published phenotype. In three cases, different dsRNA concentrations are likely to be responsible. For both, iB_03691 (*Tc-delta*) and iB_04526 (*Tc-homothorax*), lethality was observed in the screen. This was reproduced by injection into later stages and by using a non-overlapping fragment in another genetic background. In the publications, the authors mention using diluted dsRNA solutions in order to see the phenotype. Hence, it is likely that the higher concentrations of dsRNA used in the iBeetle screen caused the stronger phenotype. Pupal dsRNA injections targeting *Tc-decapentaplegic* (*Tc-dpp)* always led to lethality or cachexia (starved animals) in our screen and in follow-up experiments with two different fragments in two different strains. This is in contrast to the published results [10,11] but is in line with the recent finding that *Tc-dpp* is also involved in lipid homeostasis [12].

The timing of injection was critical for iB_01701, which targets *Tc-laccase-2*. The expected phenotype was annotated neither in the pupal nor the larval injection screen. Injection into L6 larvae reproducibly led to early lethality instead of the published tanning defect (i.e. pupae with soft and non-pigmented cuticle) while in the pupal screen, no effect was seen. An independent injection of the same fragment into older (L7) larvae did reproduce the published phenotype.

In four cases, either isoform-specific knock down, or off target effects likely led to different phenotypes. *Tc-knirps, Tc-frizzled-1* and *Tc-lim-1* were annotated with the "empty egg phenotype" instead of the published morphological defects [13–15]. These results were reproduced using the iBeetle fragment but non-overlapping fragments produced the published phenotype (*Tc-knirps* and *Tc-frizzled-1)* or wildtype larvae (*Tc-lim-1).* Pupal injection of an iB-fragment against *Tc-broad-complex* (*Tc-BRC,* iB_03960) did not reveal any defect, while injection of another fragment of *Tc-BRC* (used as a positive control) consistently led to various strong defects during embryogenesis. Our follow-up analysis indicates that off target effects are the most likely explanation: In the case of *Tc-BRC*, several splice variants with distinct functions are known for *Drosophila* and *Tribolium* and indeed, the two dsRNA fragments target different isoforms: The positive control fragment (335 bp) lies in the common fourth exon of the *Tc-BRC* isoforms as published by Konopova and Jindra (2008)[16] and leads to a range of embryonic developmental defects. iB_03960 (495 bp) does not overlap with the positive control fragment, but spans the 3' end of common exon 4, common exon 5 (adding up to 321 bp) and part of the alternatively spliced exon 6 of splice isoform Z4 (174 bp). iB_03960 does not lead to any embryonic defects. Due to the fact that both fragments are predicted to target all isoforms, an off target effect of the positive control fragment appears likely. Previous studies using an insertion mutant have shown no or no severe degree of embryonic

lethality. Due to the location of the insertion in the region of alternatively spliced exons it remains unclear what isoforms are actually affected in this mutant [16].

For *Tc-fz1* and *Tc-kni* we checked the most recent annotations of the *Tribolium* and *Drosophila* genomes and the *Tribolium* literature for indication of alternative transcripts. There are no published splice isoforms for *Tc-fz1* and off target analysis did not reveal any putative off target hit using the e-RNAi resource [17]. For *Tc-kni* there are two small 5' introns present in the automatically annotated gene model (TC003413), but not in the published mRNA sequences, which are based on RACE and cDNA sequences[14] (NM_001128495). Anyway, the iBeetle fragment targeting *Tc-knirps* is downstream of this questionable region. Off-target analysis revealed several hits in other genes two of which showed a phenotype in the screen.

One out of 65 phenotypes was overlooked (1.5%) and two were missed for technical reasons (3%). It is a bit surprising that the portion of missed phenotypes of this completely blind set of controls is even lower than the one with the set of not completely blind positive controls. The reason could be that we deliberately included subtle (partly unpublished) aspects of phenotypes as positive controls while the set of published phenotypes may be biased towards strong phenotypes.

Several RNAi phenotypes were missed. Subtle tarsal and antennal defects after *Tc-bric-a-brac* (iB_03591) larval RNAi were not detected in the screen but we were able to elicit this phenotype by repeating the experiment (with the iBeetle fragment). In two cases, technical lethality (e.g. due to injection problems) led to precocious death of the injected animals, which prohibited detection of the later phenotype (*Tc-pumilio* (iB_03898) and *Tc-methoprene-tolerant* (iB_03648)).

Negative controls

The last injection of each injection day was either a positive control or a buffer injection (blind to the screener). We used these buffer injections to assess the rate of false negative annotations: Four out of 155 buffer controls (2.6%) had false negative annotations while in another three cases technical issues led to a false positive annotation of lethality (1.9%) (see Supplementary Figure 2 for distribution of technical lethality in buffer and dsRNA injections).

Supplementary References

1. Li, J. *et al.* Odoriferous Defensive stink gland transcriptome to identify novel genes necessary for quinone synthesis in the red flour beetle, Tribolium castaneum. *PLoS Genet.* **9,** e1003596 (2013).
2. Sokoloff, A., Slatis, H. M. & Stanley, J. The black Mutation in Tribolium castaneum. *J. Hered.* **52,** 131–135 (1960).
3. Sokoloff, A. *The biology of Tribolium: with special emphasis on genetic aspects*. (Clarendon Press, 1974).
4. Pavlopoulos, A., Berghammer, A. J., Averof, M. & Klingler, M. Efficient transformation of the beetle Tribolium castaneum using the Minos transposable element: quantitative and qualitative analysis of genomic integration events. *Genetics* **167,** 737–46 (2004).
5. Prout, M., Damania, Z., Soong, J., Fristrom, D. & Fristrom, J. W. Autosomal mutations affecting adhesion between wing surfaces in Drosophila melanogaster. *Genetics* **146,** 275–285 (1997).
6. Walsh, E. P. & Brown, N. H. A screen to identify Drosophila genes required for integrin-mediated adhesion. *Genetics* **150,** 791–805 (1998).
7. Angelini, D. R., Smith, F. W. & Jockusch, E. L. Extent With Modification: Leg Patterning in the Beetle Tribolium castaneum and the Evolution of Serial Homologs. *G3 Bethesda* **2,** 235–48 (2012).
8. Angelini, D. R., Smith, F. W., Aspiras, A. C., Kikuchi, M. & Jockusch, E. L. Patterning of the adult mandibulate mouthparts in the red flour beetle, Tribolium castaneum. *Genetics* **190,** 639–654 (2012).
9. Angelini, D. R., Kikuchi, M. & Jockusch, E. L. Genetic patterning in the adult capitate antenna of the beetle Tribolium castaneum. *Dev. Biol.* **327,** 240–251 (2009).
10. Ober, K. A. & Jockusch, E. L. The roles of wingless and decapentaplegic in axis and appendage development in the red flour beetle, Tribolium castaneum. *Dev Biol* **294,** 391–405 (2006).
11. Van der Zee, M., Stockhammer, O., von Levetzow, C., Nunes da Fonseca, R. & Roth, S. Sog/Chordin is required for ventral-to-dorsal Dpp/BMP transport and head formation in a short germ insect. *Proc Natl Acad Sci U A* **103,** 16307–12 (2006).
12. Namigai, E. K. & Suzuki, Y. Functional conservation and divergence of BMP ligands in limb development and lipid homeostasis of holometabolous insects. *Evol Dev* **14,** 296–310 (2012).
13. Beermann, A., Pruhs, R., Lutz, R. & Schroder, R. A context-dependent combination of Wnt receptors controls axis elongation and leg development in a short germ insect. *Development* **138,** 2793–805 (2011).
14. Cerny, A. C., Grossmann, D., Bucher, G. & Klingler, M. The Tribolium ortholog of knirps and knirps-related is crucial for head segmentation but plays a minor role during abdominal patterning. *Dev Biol* **321,** 284–94 (2008).
15. Posnien, N., Koniszewski, N. D. B., Hein, H. J. & Bucher, G. Candidate Gene Screen in the Red Flour Beetle Tribolium Reveals Six3 as Ancient Regulator of Anterior Median Head and Central Complex Development. *PLoS Genet.* **7,** e1002418 (2011).
16. Konopova, B. & Jindra, M. Broad-Complex acts downstream of Met in juvenile hormone signaling to coordinate primitive holometabolan metamorphosis. *Development* **135,** 559–68 (2008).
17. Horn, T. & Boutros, M. E-RNAi: a web application for the multi-species design of RNAi reagents--2010 update. *Nucleic Acids Res* **38,** W332–9 (2010).
